# Revisiting estimates of diffuse contamination of agricultural soils by Pb

**DOI:** 10.1007/s10661-026-15684-5

**Published:** 2026-08-05

**Authors:** Tomáš Matys Grygar, Štěpánka Tůmová, Adéla Czolková, Stanislav Škorňa, Michal Hošek, Jitka Elznicová, Mavro Lučić, Karel Hron

**Affiliations:** 1 Institute of Inorganic Chemistry, Czech Academy of Sciences, Řež, 250 68 Czech Republic; 2 Faculty of Environment, J. E, Purkyně University in Ústí Nad Labem, Pasteurova400 96, Ústí Nad Labem, 3632/15 Czech Republic; 3 Department of Mathematical Analysis and Applications of Mathematics, Faculty of Science, Palacký University, 17. Listopadu 12, 771 46, Olomouc, Czech Republic; 4 Division for Marine and Environmental Research, Ruđer Bošković Institute, Bijenička Cesta 54, Zagreb, Croatia

**Keywords:** Soil contamination, Pb, Emission load, Geochemical data exploration, Functional data analysis

## Abstract

Knowledge of overall anthropogenic impacts on soil contamination by lead (Pb) remains insufficient despite decades of research. Estimates of diffuse Pb contamination in European soils vary by an order of magnitude. Previous research has been limited by the lack of a mathematical definition of scattered and diffuse contamination, a limited understanding of the altitudinal control of Pb emission loads, an unknown spatial extent of point contamination sources, and neglect of the natural variability of soil-forming bedrock. To address these challenges, the dataset of topsoil Pb from the Czech national monitoring and from the own soil profile sampling were processed. The soil monitoring dataset was analysed using probability density functions and functional data analysis. Altitudinal control of topsoil Pb was examined using geographically weighted and quantile regressions. Soil depth profiles from selected areas of the Czech Republic were analysed for total Pb; the stratigraphy of selected profiles was checked by gamma spectrometry. The measurable extent of point sources of Pb emission is up to ca. 30 km. Scattered contamination from more remote sources produced an altitudinally controlled increase in topsoil Pb concentrations in topographic barriers. True diffuse contamination increases with altitude and contributes between approximately 3 mg kg^−1^ at about 500 m a.s.l. and 16 mg kg^−1^ at about 800 m a.s.l. to Pb in the top 30 cm of soil, which is lower than most previous estimates. This contribution is smaller than the background Pb concentration range of 16 to 34 mg kg^−1^ and is of little toxicological concern.

**Supplementary Information:**

The online version contains supplementary material available at https://doi.org/10.1007/s10661-026-15684-5.

## Introduction

Concerns about diffuse soil contamination by risk elements such as As, Cd, Hg and Pb contributed to the recent decline of European heavy industry associated with “environmental technophobia”. Pb contamination of the Earth’s surface symbolised the negative impacts of industrialisation and insufficient environmental concern in the twentieth century (Bindler, 2011; Lau et al., 2021; Novák et al., 2003; Shotyk, 2008; Steinnes, 2009; Vácha et al., 2015). The thermal volatility of Pb (melting temperature 328 °C) favoured long-range transport of contamination (LRTC) from metallurgy, coal and waste combustion, glass and ceramic production and leaded petrol (Bińczycki et al., 2020; Fabian et al., 2017; Novák et al., 2003; Shotyk, 2008; Steinnes, 2009; Vácha et al., 2015). European ombrotrophic peat archives yielded an estimate of global anthropogenic Pb emission load of 1–10 g m^−2^ across the Earth’s surface (Bindler, 2011; Shotyk, 2008). Reimann et al. (2008a), however, argued that even the lower limit would exceed total Pb production by metallurgy in human history. A global input of 1–10 g m^−2^ would increase Pb concentrations in the upper 30 cm of topsoil worldwide by approximately 2–20 mg kg⁻^1^, whereas Fabian et al. (2017) estimated only 1–2 mg kg^−1^ diffuse enrichment in European topsoils. This order-of-magnitude discrepancy hinders objective evaluation of impacts on agricultural production safety and sustainability. The enrichment of 1–2 mg kg^−1^ is an order of magnitude below natural variability in soil composition associated with geography (e.g. southern vs. northern Europe, Reimann et al., 2012, 2018) and soil-forming bedrock (Gao et al., 1998; Vilà & Martínez-Lladó, 2015).

The scatter in estimated soil Pb emission loads may reflect undocumented local contamination sources (Bohdálková et al., 2018; Fabian et al., 2017; Hošek et al., 2024), uneven emission distribution at continental (Reimann et al., 2008a, 2008b), national (Bindler, 2011; Farmer et al., 2016) and local scales (Ahado et al., 2021; Ciarkowska & Miechówka, 2022; Szopka et al., 2013), and uncertainties in processing peat-archive geochemical data (Bohdálková et al., 2018; Twardowska et al., 2020). Proposed global impacts of regional Pb emissions (Bindler, 2011; Shotyk, 2008) is in conflict with asynchronous Pb deposition among continents (Longman et al., 2020) and heterogeneous Pb concentrations and stable isotope ratios in European soils (Reimann et al., 2012). Landscape morphology also controls spatial variability of soil Pb and other LRTC elements through interrelated factors (Ballabio et al., 2021; Skála et al., 2025; Zhang et al., 2020). Mountain barriers can receive more emissions through horizontal and wet deposition (Demková et al., 2023; Magnani et al., 2018; Szopka et al., 2013). However, altitudinal and geological controls may overlap because bedrock with larger background Pb concentrations occurs frequently in mountains (Skála et al., 2024, 2025) and along altitudinal gradients (Ciarkowska & Miechówka, 2022), potentially producing altitudinal control of geogenic background. Altitudinal control of soil risk element concentrations remains poorly understood and insufficiently incorporated into contamination mapping. Key uncertainties therefore concern the spatial extent of atmospheric contamination from point sources, historical emission sources, heterogeneity of atmospheric deposition in real landscapes and natural variability in soil composition.

Evaluating LRTC impacts requires identifying diffuse and scattered contamination, which remains undefined in exploratory data analysis for geochemistry (Matys Grygar et al., 2024, 2026; Reimann et al., 2019). Because LRTC weakly affects a considerable fraction of samples (Matys Grygar et al., 2023), it produces neither outliers defined by Tukey upper fence in boxplots (TUF) nor breaks in empirical cumulative distribution functions (ECDFs, Matschullat et al., 2000). Scattered contamination is a weak increase in target elements affecting a considerable dataset portion (e.g. the entire upper quartile Q4) and producing heavy tails in the main concentration mode (Fabian et al., 2017; Matys Grygar et al., 2024, 2026). Diffuse contamination at regional or global scale would theoretically shift entire contaminant distribution functions upward (Fabian et al., 2017; Hošek et al., 2024; Reimann et al., 2019). Distinguishing scattered and diffuse contamination is challenging but could explain the order-of-magnitude discrepancy in estimates of the Earth’s surface contamination.

This work aims to reduce uncertainties in evaluating anthropogenic contributions to soil Pb concentrations in the Czech Republic, a mid-European country with varied bedrock geology and topography and long-term human impacts on topsoils. To identify LRTC impacts and contamination extent, it first identifies historical point contamination sources. The work also corroborates the utilisation of probability density functions (PDFs) as a novel tool of exploratory functional data analysis for environmental geochemistry (Matys Grygar et al., 2024, 2026). It combines an exceptionally high-density Czech national database of HNO_3_-extractable Pb concentrations in agricultural topsoils (almost 50 thousand analyses from 54 km^2^) with sampling and analysis of soil depth profiles, mostly in hilly areas receiving the most LRTC. The soil profiles were analysed by X-ray fluorescence spectroscopy to complement acid-extractable Pb with total Pb, enabling robust estimates of topsoil enrichment independent of horizon-dependent Pb chemical reactivity. The work also examines altitude control of Pb concentrations, which remains poorly understood, and contributes to defining diffuse and scattered soil contamination in real landscapes and explaining the wide range of previous estimates of diffuse contamination in European soils.

## Study area and national soil dataset

### Study area

The Czech Republic belonged to countries with a long history of metallurgy and heavy industry (Bednářová et al., 2016; Kváčová et al., 2015; Novák et al., 2003; Vácha et al., 2015; Zuna et al., 2012) which contributed to Pb emissions. At the same time, the bedrock geology of the area is complex and includes also rocks with anomalous Pb (Hošek et al., 2024; Matys Grygar et al., 2024; Vácha et al., 2015), of which importance has earlier been underestimated. Particular contamination sources that have acted in the study area are summarised in Supplementary Information ESM1 and ESM2.

The topography of the Czech Republic and its mid-European surroundings favour uneven distribution of LRTC emissions, particularly in mountain ridges along the northern state borders. Prevailing mid-European winds are westerlies.

### Soil monitoring dataset (RKP)

National maps and density data analysis were produced from the datasets of the Registry of Contaminated Areas (RKP), in particular, its earlier part performed under national law with soil extractions by cold 2 M HNO_3_ extraction used in the years 1990–2008. In 2008 the use of cold HNO_3_ extraction was replaced by aqua regia extraction, but the coverage of the Czech area by this novel extraction is not suitable for this study. One outstanding feature of that historical dataset with cold diluted HNO_3_ was its high data density (on average, one sample was taken per approximately 1 km^2^ of agricultural soils, Matys Grygar et al., 2026). Cold 2 M HNO_3_ extracts 81% of the topsoil Pb amount obtained by actually used aqua regia method (Matys Grygar et al., 2023).

RKP, as a very valuable source of high-resolution soil mapping dataset, have already been used in several preceding research studies (Adamec et al., 2024; Bednářová et al., 2016; Matys Grygar et al., 2023, 2024; Vácha et al., 2015). The RKP dataset is suitable both for detailed maps (Bednářová et al., 2016; Matys Grygar et al., 2023; Skála et al., 2024, 2025; Vácha et al., 2015) and for exploratory functional data analysis (Matys Grygar et al., 2024, 2026). Because RKP is performed to monitor food production safety, it does not cover forested areas, which hinders the identification of contamination sources related to historical activities in the mountains, in particular those based on charcoal.

## Methods

### RKP data processing

The national dataset from RKP was separated into 77 administrative districts, as it has been shown to considerably decrease geographic data variability (narrowing too broad and featureless data distribution) and thus better define heavy tails and high outliers (Matys Grygar et al., 2024, 2026). In univariate (single element) data series, visualisation of PDFs somehow resembles performance of ECDFs (Matschullat et al., 2000; Matys Grygar et al., 2026); however, PDFs considerably expand the portfolio of functional exploratory data analysis, such as clustering or anomaly detection (Matys Grygar et al., 2024, 2026). Full mathematical procedures of PDFs construction can be found in preceding papers (Hron et al., 2016; Machalová et al., 2021; Matys Grygar et al., 2024, 2026; Talská et al., 2021).

Quantile regression (QReg) is a robust statistical method first introduced by Koenker and Bassett (1978) that models the relationship between a response variable and one or more predictor variables at different quantiles of the response variable distribution. Unlike standard regression modelling, which focuses only on the conditional mean response, QReg provides a more comprehensive view by estimating the conditional quantiles of the response variable distribution. QReg is applicable where more independent variables would be needed to describe the response of independent variables, i.e. when the desired dependence is affected by spatial noise (Ramsey et al., 2005). QReg is also less dependent on the presence of outliers, that is especially valuable in soil geochemistry, where outliers and non-normal distributions are common. In this study, quantile regression was applied at the 10th, 25th, 50th (median), 75th and 90th percentiles to capture the full heterogeneity of the altitude–Pb relationship across the distribution of Pb concentrations. Because Pb concentrations in Czech soils are highly skewed, we applied a log transformation before quantile regression analysis, which improved linearity and stabilised variance across the distribution. The subsequent statistical analyses using QReg were performed with the “quantreg” package in the R statistical software environment (R Core Team, 2023). The results were evaluated according to the statistical significance of regressions at 25th, 50th and 75th medians using *t*-test, as regressions at 10th and 90th medians were most affected by outliers.

Geographically weighted regression (GWR) was performed with ArcGIS Pro (Esri, USA). The GWR routine was set to 150 neighbours (approx. 1/5 of the average district area), logarithms of Pb concentrations were processed, and regression statistics was exported for further processing similarly as in Bílková et al. (2026) and in Matys Grygar et al. (2026). Results were visualised by showing the probability of regression between log(Pb) and altitudes from the local T-values, calculated by dividing the slope of the regression line by the error of the slope calculation. Conventional criterion was used for GWR map construction by assigning |T|> 1.98 to > 95% confidence level and |T|> 2.61 to > 99% confidence level.

### Density data analysis

The probability density functions (PDFs) represent the data distribution and are considered as relative and scale-invariant data objects, which means that each of their positive constant multiple carries the same information. These intrinsic properties of densities are reflected by the so-called Bayes space (Egozcue et al., 2006; van den Boogaart et al., 2014). Perturbation and powering are two fundamental operations in Bayes space and are defined as counterparts to the usual sum of two functions and the multiplication of a function by a scalar. Perturbing two PDFs results in their product, and powering a PDF by a constant c is the c-th power of the PDF. In practice, the densities are often transformed into usual real functions using the centred logratio (clr) transformation, which preserves the scale invariance of density data and introduces a zero integral constraint. These clr-transformed PDFs can be then analysed by the standard methods of an exploratory functional data analysis (EFDA), see Matys Grygar et al. (2024). The clr transformation is defined as the logarithm of the PDF minus its mean over the domain; the function value of the clr-transformed PDF indicates the difference between the actual and the mean (expected) log-density value, meaning that it indicates how likely the particular concentration is to occur compared to the expected log-density value.

For visualisation, the heatmaps of clr-transformed PDFs of Pb were used. Following Matys Grygar et al. (2024), the kernel density estimation was performed on the observed data (Pb concentrations) from the individual districts; then, the discretised and clr-transformed values were used for smoothing by compositional splines (Machalová et al., 2021), which were created for the basis representation of the clr-transformed PDFs and can be used in the standard EFDA methods. The resulting spline coefficients were used to perform the exploration methods presented below, i.e. the hierarchical clustering of PDFs and the functional principal component analysis (FPCA).

The aim of FPCA is to find the functional principal components (FPCs) which represent the main modes of variability in the data, the effects that contribute the most to the data variability. These are determined by orthonormal basis functions known as eigenfunctions or harmonics. The original (but centred) PDFs can be approximated by the first few principal components. The coordinates of the PDFs in the orthonormal basis are called scores and determine how each effect (eigenfunction) affects the particular PDF. Typically, the first two components are visualised to reveal major structural differences or similarities of the original PDFs; specifically, the scatter plot of the first two component scores and their corresponding harmonics is investigated. Furthermore, the percentage of explained variance by each FPC can be determined. More details can be found in Hron et al. (2016), where FPCA for density data, called simplical functional principal component analysis, was introduced. FPCA was performed on (centred) clr-transformed PDFs.

### Sampling and analysis of soil element content

Soil profiles were preferably sampled in pairs of nearby sites in forests and in agricultural fields in selected areas of the Czech Republic. Sampling was mainly performed in hilly areas with landscape mosaics of forests and agricultural fields, mainly meadows and grazing lands. In forest soils, litter (undecomposed organic detritus, L subhorizon) was removed before sampling. The top 20 cm was divided into 3 subsamples. In forest soils, F + H organic subhorizons were taken at 0-x cm (x is the thickness of the F + H subhorizons), further samples were x-10 cm, and 10–20 cm, similar to Johanis et al. (2022). In grasslands without an O horizon, 0–5 cm, 5–10 cm and 10–20 cm layers were taken in the top 20 cm. Deeper soil samples were taken at steps of 10 to 20 cm down to 70 cm or to the entire soil profile above saprolite/bedrock.

Topsoil mapping in the Lužice Mountains was performed mainly in forests and grazing soils not included in RKP. Organic detritus (litter, organic stratum L) was removed before sampling. The top 20 cm were sampled without subsoil to improve the effectiveness of pollution mapping in a larger area.

Soils were sieved with plastic mesh (< 2 mm), left to dry under ambient conditions or at 60 °C, and subsamples were pulverised in a planetary micromill. The resulting powders were analysed by X-ray fluorescence spectroscopy (EDXRF) using an Epsilon 3X spectrometer (PANalytical, the Netherlands) as reported earlier (Adamec et al., 2024; Matys Grygar et al., 2025). Signal counts were calibrated by a set of 11 certified reference materials as described in Adamec et al (2024). XRF analysis enables determination of total Pb including the refractory minerals and thus produces result better interpretable in quantitative terms of total anthropogenic contribution beside geogenic input of Pb.

Gamma spectrometry was performed using a liquid N_2_-cooled HPGe detector GCW2022-DET (Mirion Technologies, Canberra, Australia) with an efficiency of at least 20%. The gamma activity of the 2.5 cm inner layer of Pb shielding was < 5 Bq kg^−1^. Spectra were evaluated using the LYNX-MCA software (Mirion Technologies, Canberra). The detector had a well for the analysis of small samples (2–5 g), and glass vials were used as the measuring cells. The samples were kept in vials for at least 3 weeks before analysis. The ^137^Cs activity was determined from the spectral line at 661.7 keV. The background signal from the vials was below the detection limit of the detector. Determination of unsupported ^210^Pb activity ^210^Pb_XS_ was performed by subtraction of the activity peak at 46.5 keV and the activity peaks of ^214^Pb at 77.11 and 295.21 keV and ^214^Bi at 609.31, 1120.29 and 1764.49 keV, each with sensitivity ratios of the ^210^Pb and all listed 214 activity peaks empirically adjusted to the background samples as described in Matys Grygar et al. (2022). ^137^Cs and ^210^Pb certified reference materials in the same vials were provided by the Czech Metrology Institute (Prague, Czech Republic).

Fallout input of ^137^Cs (mainly from the Chernobyl nuclear accident in 1986) to topsoils was uneven within more than an order of magnitude topsoil surface activity due to irregular distribution of Chernobyl contaminants spread over Europe and uneven precipitation early after the accident (Suchara, 2017). Fallout input of ^210^Pb_xs_ should be more evenly distributed as it originates from radon decay of ^222^Rn in the atmosphere. However, activities above the detection limit were found in all topsoils of undisturbed soil profiles. ^210^Pb_xs_ is mainly caught in organic topsoils, while ^137^Cs tends to be translocated to mineral-containing soil horizons, where it is sorbed to clay minerals (Baccolo et al., 2023).

Average Pb concentrations in the top 30 cm were calculated as depth-weighted means of individual sampled soil horizons from that interval. Total pools of Pb in soil profiles were estimated from Pb concentrations above the actual local background in each profile found in depth below 50 cm in the same profile to eliminate the impact of variable Pb concentration in bedrock. In shallower soil profiles, the background was taken from the nearest site with the same background geology. Soil density was estimated as 1.0 g cm^−3^ for F + H organic horizons and 1.6 g cm^−3^ for mineral horizons.

### Use of AI tools

OpenAI’s ChatGPT (GPT-5.5 Thinking; accessed on 24th June 2026) was used to assist with language smoothing and condensation of the Introduction and selected parts of the Discussion. The tool was instructed to reduce redundancy and improve conciseness while preserving the original meaning, scientific terminology, numerical values and references. All suggested revisions were subsequently reviewed, corrected where necessary, and approved by the authors. ChatGPT was not used to generate anything relevant for this work. The authors of this work take full responsibility for all possible errors.

## Results

### Contamination shown in the RKP concentration maps

**Fig. 1:**
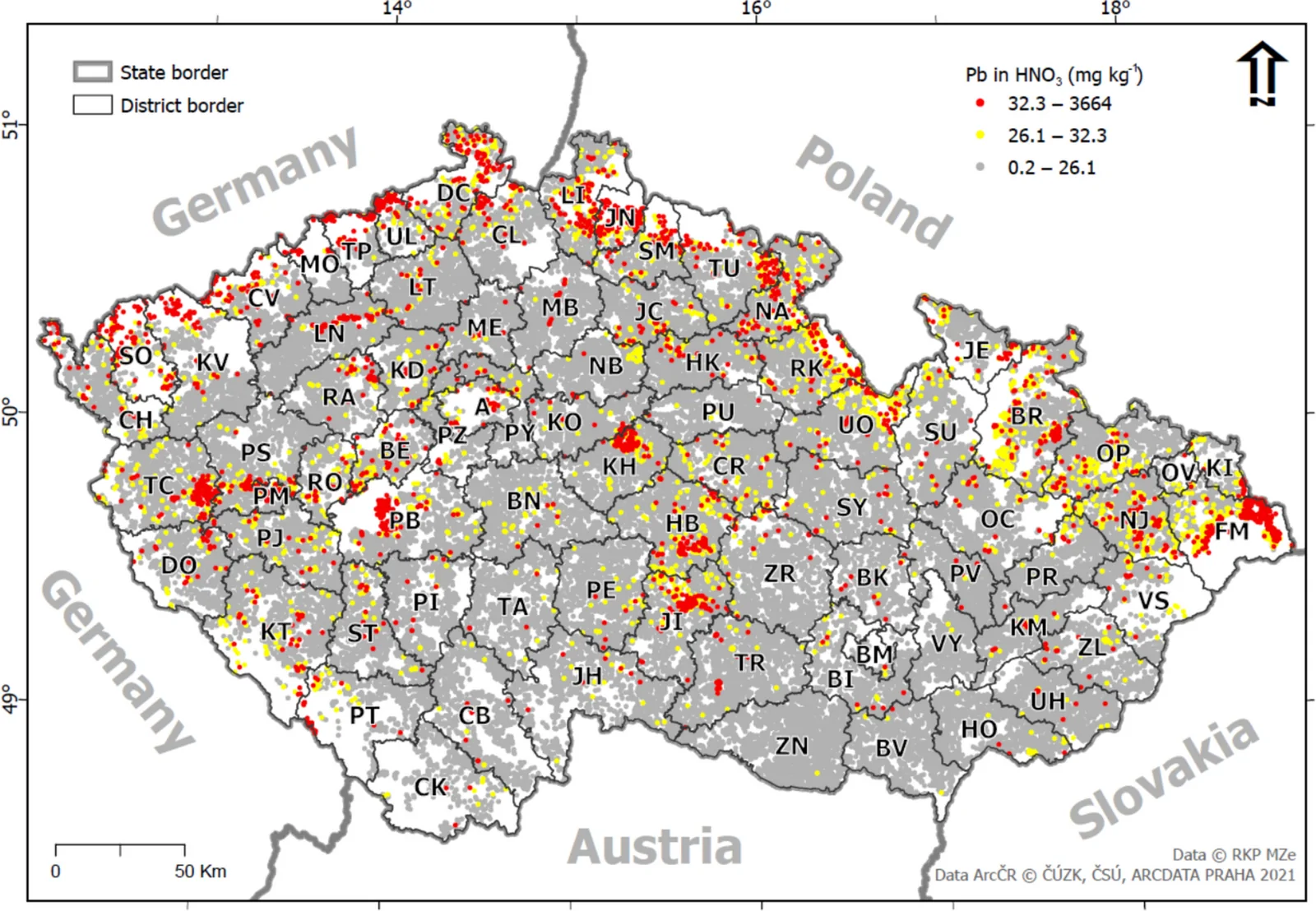
Map of high outliers (red circles) and elevated values (yellow circles) of HNO_3_-extractable soil Pb from the RKP soil monitoring. Two-letter labels and letter A denote administrative districts of the Czech Republic

The national map of HNO_3_ extractable Pb in agricultural topsoils is shown in Fig. 1. The map shows where Pb concentrations exceed the TUF-based threshold calculated for the entire data series, i.e. univariate Pb outliers (red circles in Fig. 1). Fig. S1.1 in ESM1 shows geographic hotspots of the Pb soil concentrations in an alternative manner of Getis-Ord geostatistics. Both 1 and Fig. S1.1 visualise the most influential point contamination sources, most of which have been known and are summarised in ESM1. The majority of localised contamination hotspots (point contaminations) are around historical centres of Ag and Pb metallurgy (Table S1.1 in ESM1); they are typically isometric (circular or elliptical), with a typical diameter of up to 5 to 10 km. One larger and less isometric hotspot of soil Pb is in the north east of the Czech Republic, located around iron- and steel metallurgy centres, whose emissions have been captured by slopes of the Beskid Mountains and thus impacted practically the entire FM district (Fig. 1).

The shape of further larger anisometric hotspots can be a consequence of three possible factors: (i) bedrock geology with elevated background Pb, (ii) geomorphology, such as river valleys or mountain ranges, which have accumulated historical contamination from more remote sources or (iii) a combined impact of nearby historical sources, including unknown ones. Bedrock geology with frequent ore veins and historical metallurgy in the Ore Mountains (along the northern part of the Czech-German border, Hošek et al., 2024; Fig. S1.1 in ESM1) contributed to high soil Pb in the north-west of the Czech Republic (NW parts of SO, KV, CV, MO, TP and UL districts, Fig. 1). The strip with elevated soil Pb concentrations in BR, OP, NJ and PR in the Jeseník Mountains is associated with local Palaeozoic marine sediments deposited under volcanic influence (Matys Grygar et al., 2023). The majority of linear hotspots are in floodplains of meandering rivers with broad alluvial floodplains, such as the Ohře River, draining several historical ore regions where mining culminated in the sixteenth century (LN and LT districts, line of red circles in LN district in Fig. 1, hotspot 25 in Fig. S1 in ESM1). Other floodplain hotspots are specified in ESM1. Floodplain hotspots have been formed in the first fluvial depositional areas downstream of contamination sources and incised river channels with very limited floodplain deposition.

Topsoils with elevated Pb are also along the northern border of the Czech Republic, but here the reasons are less clear. Several areas can be distinguished: the northern part of DC and CL districts, the area from LI and JN to the SM districts, and the mountain slopes along the Czech-Poland border in a strip through TU, NA, RK and UO districts (Fig. 1 and in S1.2 in ESM1). These northern boundary mountain slopes have traditionally been considered by local researchers to be impacted by LRTC from the heavy industry of the second half of the twentieth century. The hotspot in the boundary of DC and CL districts has been subjected to detailed topsoil mapping (Fig. S2.2 to S2.4 in ESM2) to fill the “white spot” in the RKP topsoil map (Fig. 1 and Fig. S2.1 in ESM2) around the northern boundary between those districts: the Lužice Mountains. The strip from TU to UO could be a candidate for an area impacted by scattered contamination by LRTC.

Remarkable in Fig. 1, as well as in Fig. S1.1 in ESM1, is a smaller portion of topsoil Pb outliers in the southern part of the Czech Republic in districts remote from known point contamination sources. Particularly low number of outliers is in districts CK to BV along the boundary with Austria. Also noteworthy is the very low presence of outliers in lowland agricultural districts such as, e.g. LT, ME, NB and PU (Fig. S1.2 in ESM1).

### Contamination shown in RKP density data

**Fig. 2:**
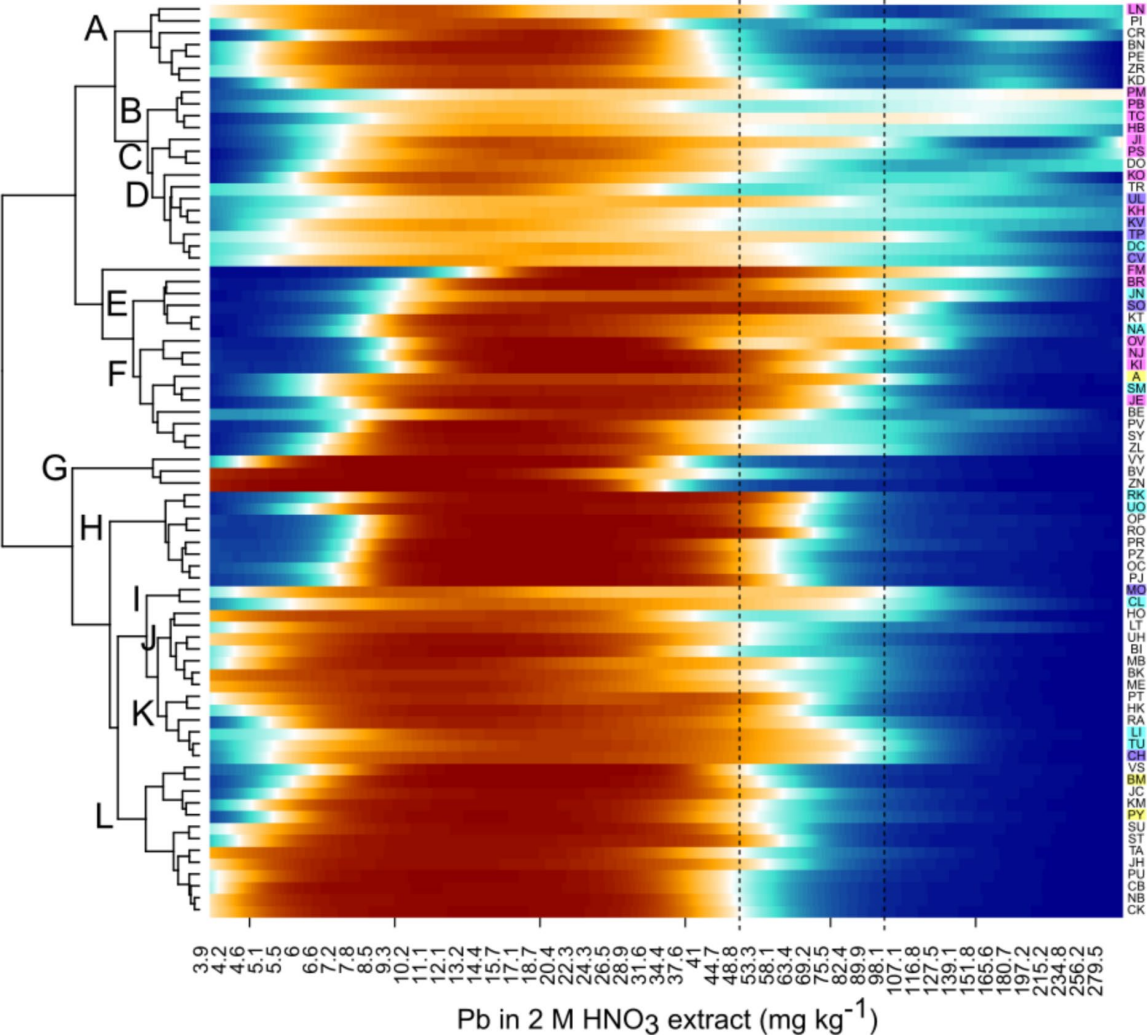
PDFs show the relative density distribution of soil Pb concentration in each district along the concentration axis. The high probability density is marked by warm (red) hues, while cold (deep blue) hues show small probability densities (they are negative after the clr transformation). The scale of clr PDFs is relative and amplifies density heterogeneity in heavy tails and around edges of the distributions

Soil contamination can be visualised by changes in the shape of PDFs of Pb, as for individual administrative districts shown in Fig. 2. The features of the heatmap compared to traditional visualisation of data distributions can be found in Matys Grygar et al. (2026). The heatmap displays probability densities in a colour scale with high-density values in warm (red) hues and low-density values in cold (blue) hues. The more intensive the colour, the greater the difference between the log-density and the expected one. Those colours thus depict the position of the main concentration modes with high probability densities in warm colour hues (toward red) roughly in/left of the central part of rows, as well as the character of edges of the main mode. Sharp colour contrasts on the arms of the main mode indicate narrow distribution of the main mode (e.g. in clusters H and L), while the weaker colour contrast visualises heavy tails (e.g. in clusters B and D) and possibly also high outliers (if present) as locally warmer colours above the main concentration mode (e.g. LN and CR in cluster A and LN in cluster B). Districts are clustered according to similarity in their PDFs on the left side of Fig. 2, which was done using the standard hierarchical clustering method with complete linkage criterion, starting from the matrix of L^2^ distances between real functions (clr-transformed PDFs). The number of clusters is selected by a balance of visual assessment and geological expertise, i.e. with the use of interpretability criteria. The position of the main concentration mode in the districts in Fig. 2 can show varying background concentration in districts: generally, higher Pb concentrations in the main mode are in districts in clusters E, F and H, while low concentrations in the main mode are in districts in clusters G, J and part of L. The low main modes are typical for districts in lowlands with sedimentary rocks as soil-forming substrates with large coldspots in Fig. S1.1 in ESM1.

Severe point contamination, i.e. a small fraction of samples with very high Pb concentration, produces heavy tails or outliers at the right arm of the main mode and thus decreases colour contrasts at high concentrations above 50 and 100 mg kg^−1^ (districts in clusters B, C and D in Fig. 2). Those clusters contain most districts with severe point contamination by non-ferrous metallurgy (hotspots in Fig. 1, Fig. S1.1 and list in Table S1.1 in ESM1); in Fig. 2, those heavy tails and high outliers “smear” the main mode (orange hues between approximately 50 and 130 mg kg^−1^). Those clusters also include districts with geogenic anomalies/historic metallurgy in the Ore Mountains (strip of districts from SO to UL).

Districts CL, LI and TU (clusters I and K in Fig. 2) have all smeared main modes and heavy tails at high concentrations (low colour contrasts around 50 and 100 mg kg^−1^, indicative of some local contamination). Because there were neither metallurgy nor geogenic anomalies in those districts, we searched for their cause. Soil mapping was performed in the forested area of the Lužice Mountains around the borders of districts DC and CL underrepresented in RKP (Fig. S2.1 in ESM2). It revealed several local Pb hotspots around centres of historical production of decorative glass (Fig. S2.2 in ESM2). This area belongs to the “glass belt” spanning from the Lužice Mountains (from east part of DC through the northern part of CL and LI and JN to the northern parts of SM districts (Fig. S2.1 in ESM2).

The districts with spatially more extensive (wider areal, scattered) contamination and without high outliers have quite specific PDFs. For example, the entire main mode of the FM district (the single line between clusters D and E in Fig. 2) is shifted to higher concentrations relative to all other districts because of the massive impact of iron- and steelmaking NW of the hotspot in FM (Table S1.1 and Fig. S1 in ESM1). The main mode in the FM district has a very sharp left arm, showing that this district contains a smaller fraction of low-Pb samples than any other district, i.e. its pattern is typical for relatively weak contamination in terms of concentrations but massive in terms of fraction of samples impacted (Fabian et al., 2017). Relatively low density of samples with Pb concentrations < 10 mg kg^−1^ (absence of low-Pb samples) was also found in districts JN, SM, NA, RK and UO (clusters E, F and H) from the strip along Czech-Polish boundary; their entire main mode was elevated relative to the rest of the Czech Republic (Fig. 1) as well as relative to their surrounding (Fig. S1.1 in ESM1), but the PDFs show rather sharp right arm, i.e. no heavy tails and high outliers (Fig. 2). It could indicate that either LRTC affected entire districts, or elevated background in those districts; those two options were examined by soil depth profiles (below). The soil Pb hotspots along the Czech-Polish boundary in Fig. 1 thus represent just the right part of their main modes rather than severe contamination from point sources.

The majority of districts in clusters G, J and L appear not contaminated relative to other districts according to their PDFs (sharp main modes, low concentrations in main modes, no heavy tails, Fig. 2). The districts are mainly in lowlands without local point sources and in the southern part of the Czech Republic, both of which showing Pb concentration coldspots in Fig. S1.1 in ESM1. In those districts, scattered contamination by LRTC was absent or particularly weak.

**Fig. 3:**
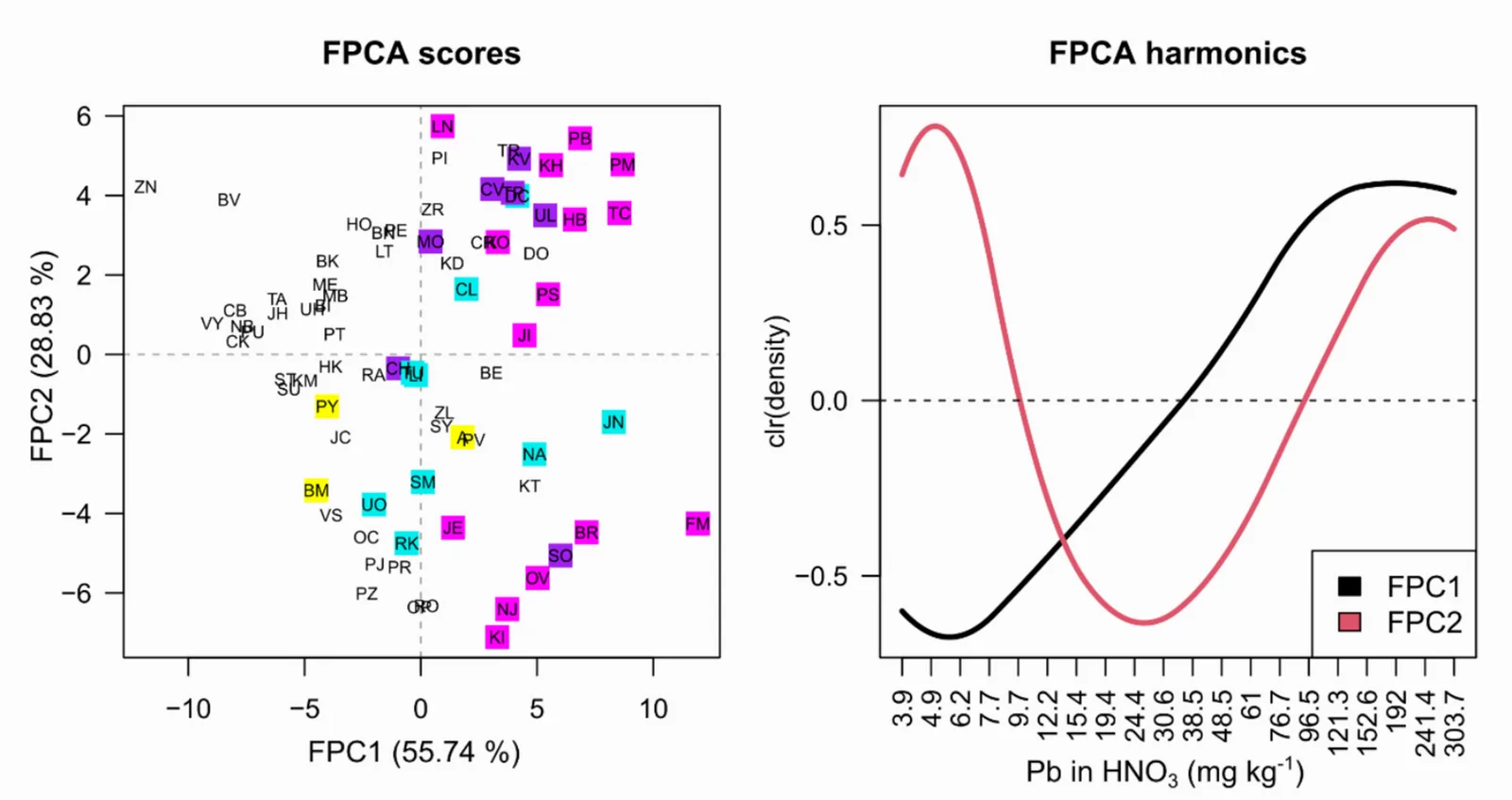
The first two functional principal components of the density distribution of soil Pb concentration in each district. Colour codes of districts as in Fig. 2. FPC1 represents the location of the main mode of Pb concentrations: the more contaminated districts, the higher FPC1 score values. FPC2 captures the occurrence of heavier tails in Pb densities: districts with positive FPC2 scores have distributions with heavier tails. A zero score means that the district’s PDF does not differ from the mean (expected) distribution along the corresponding FPC

PDFs can also be processed by FPCA (Fig. 3). The scores in Fig.  3A show the coordinates of individual districts corresponding to the two functional principal components (FPCs) represented by the curves in Fig. 3B. These components represent the main patterns of variability in the data—that is, the effects that contribute to 86% variability in the data and to the differences in concentration distributions across districts. FPC1 represents a more likely presence of higher Pb concentrations. Districts with positive FPC1 scores have heavier right tails, which is visualised in the heatmap (Fig. 2) as decreased colour contrasts at right arms. The districts contaminated by smelting are thus plotted on the right side of Fig. 3A. Conversely, in districts with negative FPC1 scores, higher Pb concentrations do not occur, and they have dark blue right tails in the heatmap. FPC2 represents the occurrence of both very low and very high Pb concentrations. Districts with positive FPC2 scores thus have broad distributions with heavier tails, and the variance of the measured Pb concentrations is greater. Conversely, districts with negative FPC2 have narrower distributions, the variance of measured concentrations is smaller, and very low and very high concentrations occur less frequently in these districts.

Specific combinations of the first and second components separate districts around short-range (local) point contamination sources around historical Ag metallurgy centres (HB, KH, PM, PB, TC) with more frequent high outliers and a considerable part of the main mode with low Pb, thus with positive FPC1 and positive FPC2 (top right in Fig.  3A), which are in clusters B and C in Fig. 2. Districts, in which contamination is more massive due to LRTC manifested by a heavy right tail rather than outliers, have positive FPC1 and negative FPC2 (Fig.  3A) and include the most severely impacted district FM and districts in cluster D (Fig. 2). Noteworthy are the districts plotted diagonally along the lower left edge of the cloud of points in Fig.  3A, i.e. along the line from ZN through VY and VS to OP, have the lowest portions of high outliers, the least heavy tails and the main mode with gradually growing overall Pb concentration from ZN to OP. Accordingly, the districts along that diagonal mostly belong to clusters G and H in Fig. 2 with very low probability densities at right arms. These districts are the least impacted by local contamination and are candidates for the determination of diffuse contamination.

### Contamination visualised by altitudinal control of RKP concentrations

**Fig. 4:**
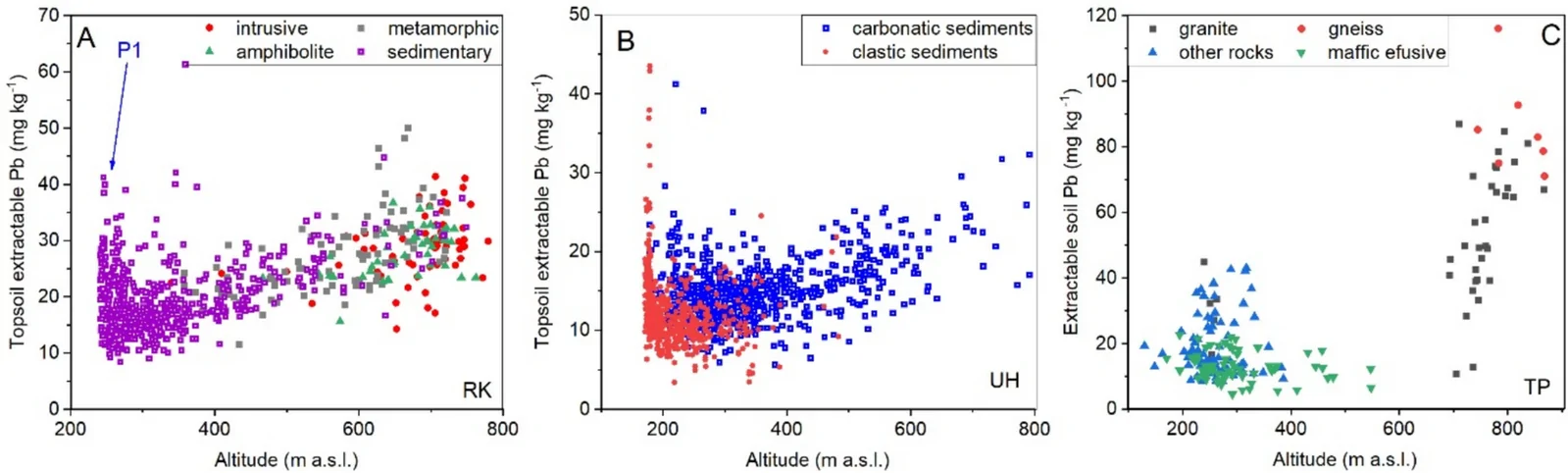
Examples of altitudinal patterns P2 (real altitudinal control) for RK district (**A**) and UH district (**B**), and pattern P4 (geological artefact) for district TP (**C**). P1 pattern is topographic artefact related to a local floodplain

**Fig. 5:**
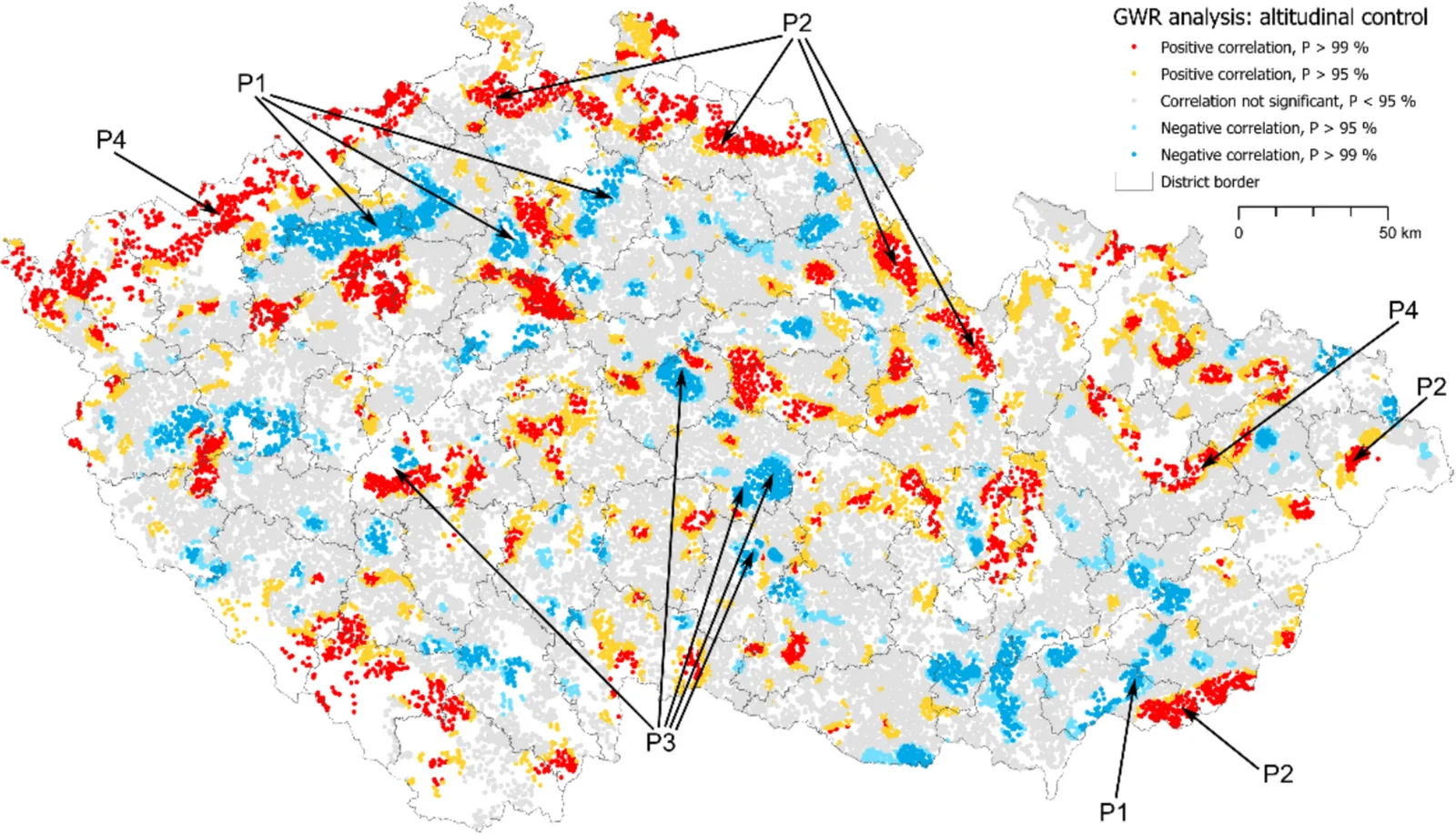
Strength of ln(Pb) regressions with altitude evaluated by GWR with 150 neighbours. Red colours show positive correlations, blue colours show negative correlations, and legends show probability levels of correlation. Negative correlations are common along floodplains of lowland rivers, scavenging catchment contamination, while positive correlations are common along topographic barriers, scavenging LRTC. P# refers to patterns listed in Table 1

QReg revealed that topsoil Pb concentration depends on altitude in the majority of districts in the Czech Republic as shown in ESM3. Statistically significant altitudinal control was thus found in 53 out of 77 districts, 8 negative correlations and 45 positive correlations (Table in ESM3). The mean number of points in one district is 700, and the largest districts are represented by more than 1000 samples in RKP; thus, the entire district is rather large, and the whole-district scale is spatially rather coarse. The altitudinal control of topsoil Pb was thus examined additionally at a finer spatial scale using GWR. The map in Fig. 5 shows probability and sign of ordinary linear regression of ln(Pb) and altitude for a sliding window with 80 neighbours, i.e. at a scale of approximately 1/10 of the whole district per run. GWR confirmed quite widespread altitudinal control of topsoil Pb (Fig. 5). The districts with significant altitudinal control were then examined individually using information on the district geography and bedrock geology; the results are summarised in Table 1 and show several distinct causes, both anthropogenic and natural. In some cases, the altitudinal control results from Pb-richer bedrock geologies exposed at higher altitudes (Fig. 4). Five typical patterns of altitudinal control labelled P0 to P4 were distinguished at the district level (Table 1): no altitudinal control (P0), soil Pb growing with decreasing altitude along broad alluvial plains (fluvial anticorrelation, P1), soil Pb growing with growing altitude with no interference from local contamination and geology (genuine altitudinal correlation, P2, Fig.  4 A and B), interference from local contamination (topographic artefact, P3), and interference from local geology (geological artefact, P4, Fig.  4 C). Topographic artefacts around point sources (pattern P2) are found around all non-ferrous metallurgy centres (Table S1.1 in ESM1), not situated in a really flat landscape. Artefacts related to the bedrock geology (pattern P3) can be demonstrated in the districts, including the Ore Mountains or the Jeseník Mountains in the (Fig. 5) as described above.

**Table 1:** Patterns of altitudinal control at the district level

Pattern	Type of dependence	Cause of the dependence		Districts
P0	No altitudinal control			CB, CK, DO, DC, HB, HK, KI, KO, ST, PY, PZ
P1	Anticorrelated	Contaminated alluvial plains		LN
P2	Correlated: emissions caught on topographic barriers	Scattered contamination from proximal sources	Iron and steel production, Beskid Mts	FM, NJ, OP
			Districts east of Ag/Pb metallurgy	BN, ZR
		LRTC from remote sources	Northern Sudetic mountains	LI, JN, SM, TU, NA, UO, RK
			Carpathian Mts	VS, ZL, UH, HO
			Vysočina Mts	JH, BE
P3	Variable: topographic artefact	Point contamination sources in undulated topography		JI, KH, PB, TC
P4	Correlated: geological artefact	Geology/metallurgy at a higher altitude	Ore Mts	KV, SO, MO, CH, TP, UL
		Geology (metallurgy) at a higher altitude	Culmian strata Jeseník Mts	BK, PV; OC, PR, NJ
			Other	TR
	Correlated	Unclear		LT, ME, NB, JE, KD, KT, OC, SY

Most pronounced genuine altitudinal control (pattern P2) was found in districts with mountain ranges acting as local topographic barriers to the prevailing westerly winds, with the nearest Pb emission sources in districts westwards. The strip between CL and UO districts along the Czech-Polish border with elevated soil Pb (Fig. 1 and S1.2 in ESM1) also shows significant altitudinal control of Pb (Fig. 5, Table 1). Figure 5 also show altitudinally controlled Pb contamination along the southern part of the Czech-Slovak border in BV, UH and ZL districts, although there is no topsoil Pb hotspot there in the national scale (Fig. 1) and BV, UH and ZL belong among districts with low overall soil extractable Pb (clusters G and J in Fig. 2 and position close to the left bottom diagonal in Fig. 3).

### Contamination in soil depth profiles

**Fig. 6:**
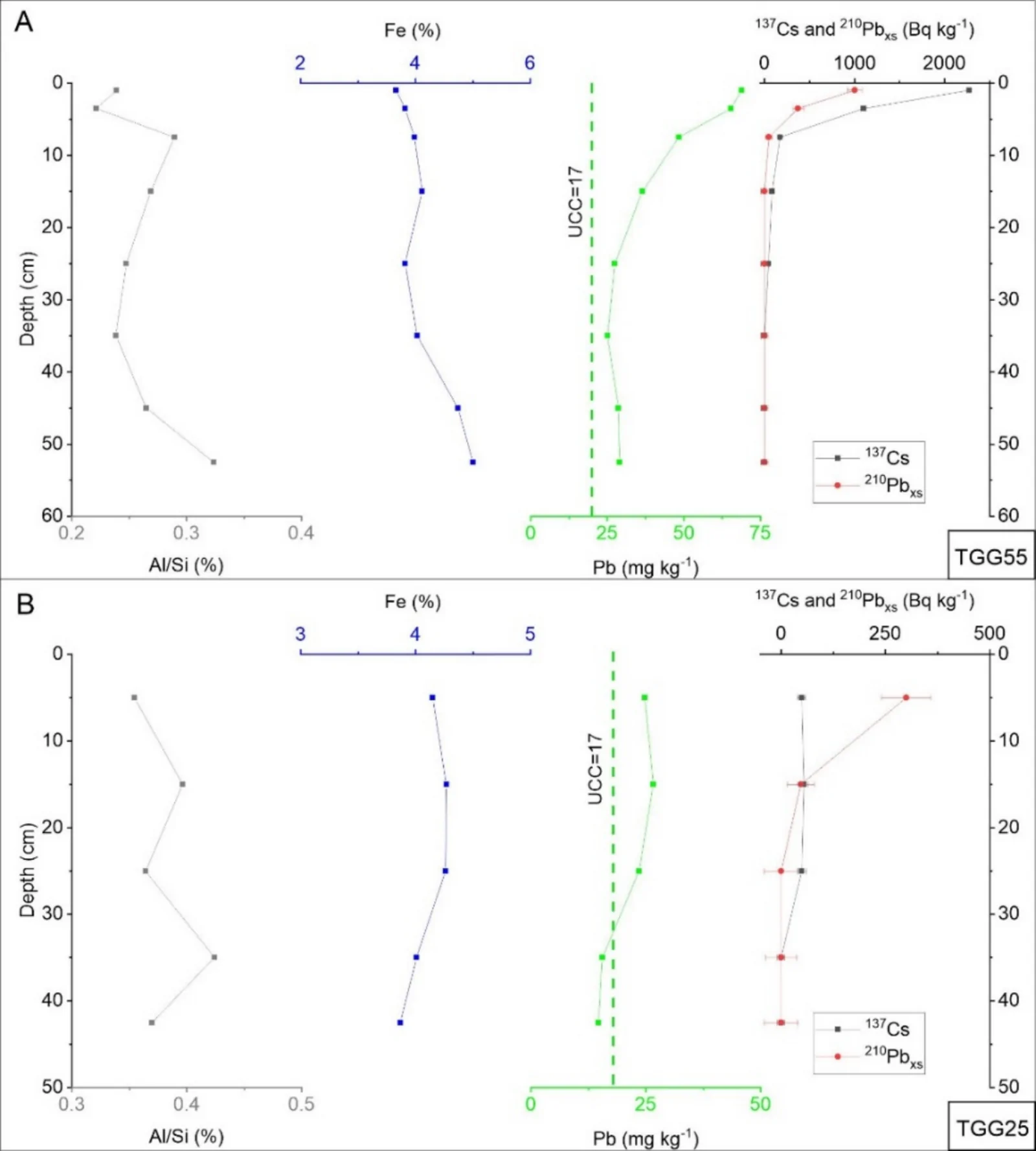
Selected depth profiles of major element characteristics, total Pb and fallout radionuclide activities in forest (**A**) and grassland (**B**) soils. Position of profiles is shown in Fig. S4.1 and S4.3 in ESM4

Soil depth profiles were obtained from forest and agricultural soils in representative areas of the Czech Republic. Maps of the areas of sampling are shown in ESM4. In the profiles, the Al/Si serves as a proxy for grain-size, and this together with Fe are good measures of soil matrix: very low Al/Si and Fe would show too high quartz content and thus insufficient soil sorption capacity to retain emission loads. Low Fe at high Al/Si would indicate organic-rich soils or soil horizons, with good sorption capacity for Pb and ^210^Pb_xs_ but low capacity for ^137^Cs. The fallout radionuclides were analysed in selected profiles to check their soil stratigraphy (Fig. 6 and ESM5). In the majority of forest soils, total Pb concentrations as well as ^210^Pb_xs_ increased upwards to their sharp maxima in organic horizons F + H, while ^137^Cs activities have their topsoil maximum somehow smeared by downward migration (Fig.  6A and S5.1 in ESM5). In agricultural soils, the topsoils showed an average by tillage with a rather step-like maximum of Pb and both fallout radionuclides on top 30 cm (Fig. 6B), sometimes tillage decreased ^210^Pb_xs_ below determination limits. Gamma spectrometry confirmed several types of disturbances, of which the most relevant for this work were (i) accumulation of the emission loads in extraordinarily thick top organic horizons (Fig. S5.2 and S5.3 in ESM5) and (ii) possible erosional removal of emissions in meadow soils, which affected both total Pb concentrations and fallout radionuclide activities.

**Fig. 7:**
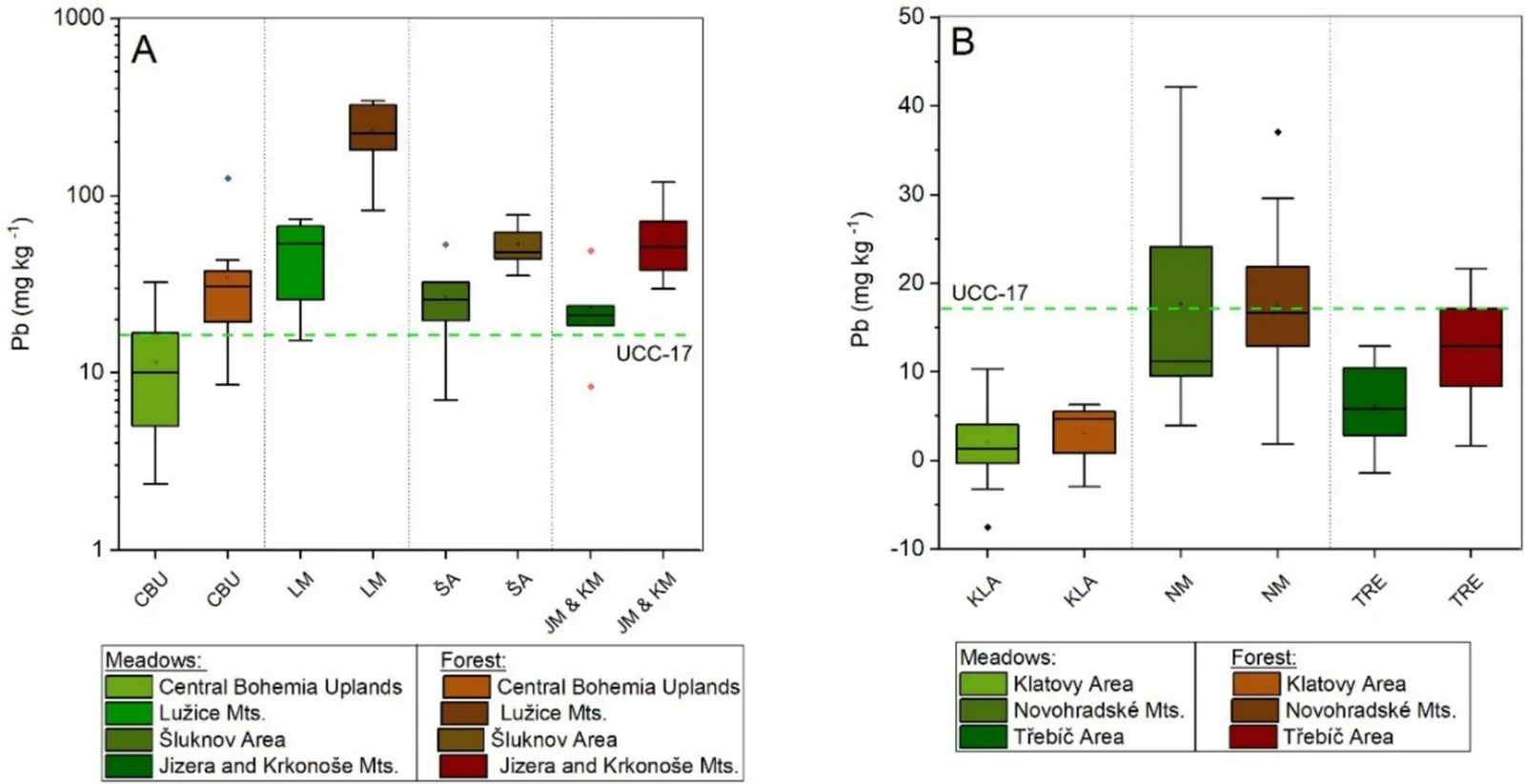
Difference between total concentrations of Pb in the top 30 cm and bottom soil strata in forest and agricultural soils in northern (**A**) and southern parts of the Czech Republic (**B**). The log concentration scale was used in panel A, showing remarkably higher Pb contamination

In stratigraphically undisturbed forest soils, a major part of Pb contamination has been focussed in the top centimetres of organic topsoils (Fig.  6A). To compensate for the effect of tillage for comparison of forest and agricultural soils, topsoils were compared after averaging Pb concentrations in the top 30 cm. Local contamination was then expressed as the difference between Pb concentrations in the averaged topsoil and bottom strata (Fig. 7), which also eliminates the effect of locally variable background. Figure  7 A shows considerably larger contamination in the northern part of the Czech Republic and more contamination caught by forest soils relative to agricultural soils. The most contaminated topsoils were found in the Jizera and Lužice mountains (Fig. S5.2 and S5.3 in ESM5), while much less in the southern part of the Czech Republic (Fig.  6A and S5.5 in ESM5). The topsoil enrichment and differences between forest and agricultural soils are much smaller in the generally less contaminated southern part of the Czech Republic (Fig. 7B).

**Fig. 8:**
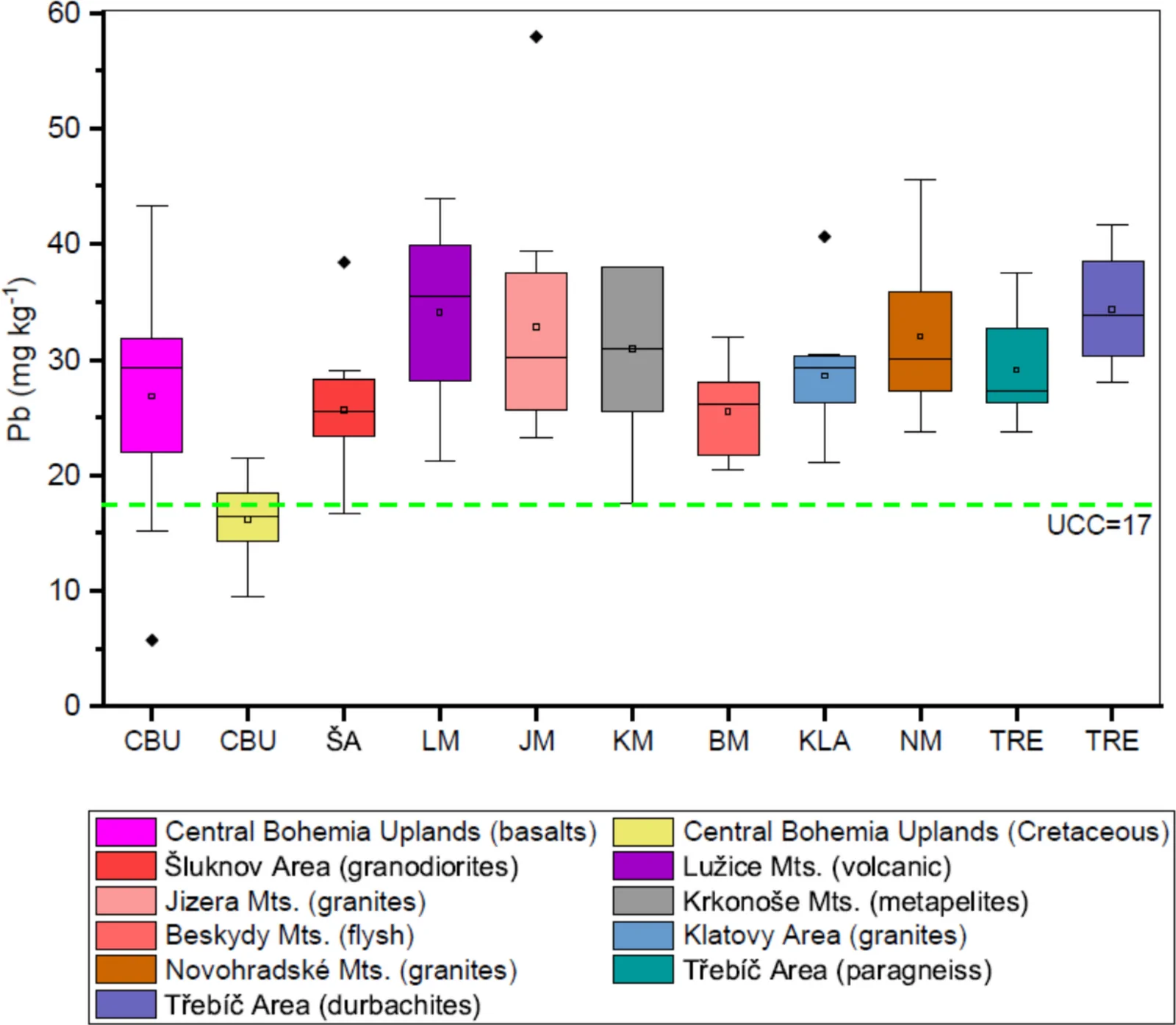
Background Pb concentrations in subsoils post-stratified according to geographic position (maps in ESM4) and bedrock geology

Subsoil Pb concentrations from depth profiles are shown in Fig. 8 and document natural variability due to local bedrock. Neither ore regions nor geogenically anomalous areas were sampled for that overview, but the background soil Pb is larger than average upper crustal rocks. The subsoils on the Cretaceous sediments contain about half the Pb (median of 16 mg kg^−1^) compared to soils on other rocks (medians of 25 to 35 mg kg^−1^). There is no systematic difference between geogenic soil Pb on felsic volcanic and metamorphic rocks in the southern and northern parts of the Czech Republic (except for the anomalous soils in the Ore Mountains along the north-western Czech state boundary), which confirms the anthropogenic cause of higher topsoil Pb concentrations in the northern part of the Czech Republic (Fig. 1).

## Discussion

### Indispensability of more ways to identify soil contamination

Conventional geochemical maps showing Pb concentration maxima (Ballabio et al., 2021; Bednářová et al., 2016; Matys Grygar et al., 2023; Reimann et al., 2012, 2018; Sucharová et al., 2011) or exceedance of legislative limits (Tóth et al., 2016; Vácha et al., 2015) are indispensable for initial soil data exploration, but cannot distinguish natural variability from anthropogenic impacts (Matys Grygar et al., 2023; Reimann et al., 2012, 2018) or reveal weak and scattered contamination at lower levels (Fabian et al., 2017; Matys Grygar et al., 2023, 2026; Reimann et al., 2019). Such maps (Fig. 1) and hotspot analysis (Fig. S1.1) identify point contamination exceeding total or local variability. Moving-window calculation of concentrations above TUF improves detection of point contamination above local variability (Skála et al., 2024), but still primarily identifies high outliers. Scattered contamination, dispersed to more distant areas and “diluted” by distance, may not produce outliers but can appear as heavy tails or related changes in PDFs for soil-data subsets (Matys Grygar et al., 2024, 2026). PDFs visualise heavy tails irrespective of main-mode position (Figs. 2 and 3). Main-mode position may reflect variable background Pb controlled by soil-forming bedrock (Appleton & Adlam, 2012; Vilà & Martínez-Lladó, 2015; Gosar et al., 2019; Matys Grygar et al., 2023; Skála et al., 2025; Fig. 8), thereby partly accounting for natural variability at district level.

Genuine diffuse contamination affecting almost an entire district is weaker, with anthropogenic additions approaching natural soil variability. Detecting such large-area contamination requires soil depth profiles (Farmer et al., 2016) or at least paired topsoils and subsoils (Fabian et al., 2017; Hošek et al., 2024; Reimann et al., 2019). Element depth profiles (Fig. 6 and ESM5) are thus preferred for identifying variable local background (Fig. 8), although geological control is essential for delineating LRTC extent, but remains rarely considered in contamination mapping and quantification.

LRTC Pb load to Czech soils frequently increases with altitude (ESM3 and areas with positive correlations on topographic barriers in Fig. 5). The geological bias must be considered (Table 1): true altitudinal correlation must occur within all individual local bedrock units, such as RK and UH (Fig.  4 A and B). The so observed altitudinal control agrees with previous reports of increasing soil contamination with elevation (Ancora et al., 2021; Ciarkowska & Miechówka, 2022; Korzeniowska & Krąż, 2020; Matysek et al., 2008; Steinnes, 2009; Szopka et al., 2013). Altitudinal dependence may therefore identify LRTC in areas remote from point contamination sources. However, altitudinal control of topsoil Pb may also reflect anomalous rocks exposed in mountain ranges (Ciarkowska & Miechówka, 2022; Skála et al., 2024, 2025), represented by pattern P4 in Table 1. Topographically variable diffuse contamination therefore cannot be approximated by a constant added to subsoil ECDF to fit topsoil ECDF, as in Fabian et al. (2017) and Reimann et al. (2019); such forced constant would underestimate diffuse contamination.

**Fig. 9:**
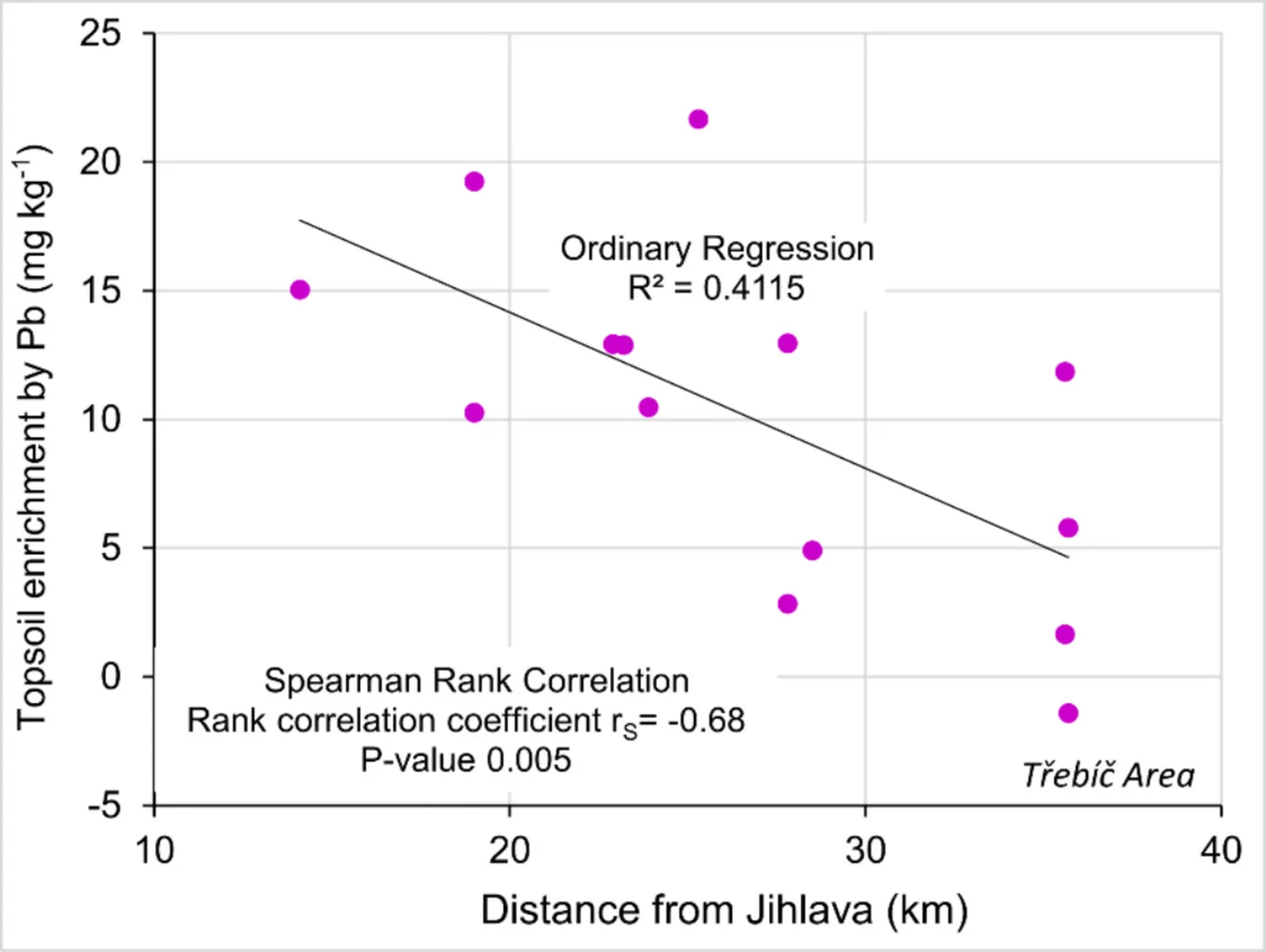
Topsoil enrichment by Pb in the Třebíč Area plotted against distance to the nearest historical Ag smelter in Jihlava

Diffuse atmospheric contamination would be overestimated if deposition archives used to reconstruct past Pb emission loads included scattered contamination from unknown historical sources. The distance over which scattered contamination exceeds natural topsoil variability remains unknown. Relatively small historical (mediaeval and early modern) smelters (Fig. 1, Fig. S1.1 in ESM1) caused point contamination (soil Pb outliers) within 5 to 10 km. Larger modern ferrous metallurgy (more pollution and higher chimneys) produced national-level Pb concentration outliers across most of FM within ca. 20 km; contamination was efficiently scavenged by the Beskid Mountains’ slopes (Bílková et al., 2026; Matys Grygar et al., 2023; Matysek et al., 2008). Scattered contamination, expressed as heavy tails but not necessarily outliers, can extend even farther than 20 km and be captured at the nearest topographic barrier in the prevailing wind direction. In TR, heavy tails and ca. 1% high outliers are attributable to JI emissions (Fig. 1), because TR has neither geogenic Pb anomalies nor historical mining or smelting. Its PDF resembles those of other smelting-affected districts (Figs. 2 and 3). Soil depth profiles show topsoil enrichment decreasing with distance from the historical smelting centre in Jihlava (Fig. 9); linear extrapolation indicates negligible impact beyond 40 km (regression line in Fig. 9).

Geochemical maps show scattered topsoil Pb contamination on topographic barriers in RK and UO (Fig. 1, Fig. S1.1 in ESM1) at slopes of local mountain range. The nearest historical sources are 50 to 100 km west; however, this mountain range forms the first topographic barrier east of those sources. The capture of LRTC is also evident from increasing soil contamination with altitude in RK and UO (Figs. 5 and 4). LRTC elevated Pb across both districts without pronounced heavy tails because whole-area contamination keeps the main modes sharp (Fig. 2, cluster H, and the position near the diagonal lower edge of the point cloud in Fig. 3). This pattern matches ECDF changes caused by diffuse contamination described by Fabian et al. (2017) and Hošek et al. (2024). Such long emission transport can occur over flat landscapes: Bindler (2011) detected impacts of the Rönnskär smelter 80 km away in northern Sweden. Transport over hundreds of kilometres has been proposed from England to Scotland (Farmer et al., 2016) and Norway (Steinnes, 2009), but these interpretations require critical evaluation, as do historical estimates of total global Pb emission loads to Earth’s topsoils.

### Underestimated and overestimated causes of elevated topsoil Pb

Many reports of Pb contamination of European soils by LRTC written from the turn of the twentieth and twenty-first centuries analysed forest floors and peat cores in mountainous areas (Steinnes and Njåstadt 1995; Novák et al., 2003; Shotyk, 2008; Bindler, 2008; Sucharová et al., 2011; Szopka et al., 2013; Johanis et al., 2022; Michczyński et al., 2022). Higher topsoil Pb in mountainous areas reflects altitudinal control (Fig. 5) and greater scavenging of LRTC by forests than by agricultural fields (Fig. 7). Czech mountain forest soils typically contain around 80 ppm Pb in their few-centimetre-thick O horizons (Johanis et al., 2022; Sucharová et al., 2011), decreasing to a median of 27 ppm at 20-cm depth (Sucharová et al., 2011). Similar concentrations occur in mountain organic soil horizons in southern Norway (Steinnes & Njåstadt, 1995), Poland (Ciarkowska & Miechówka, 2022; Michczyński et al., 2022; Szopka et al., 2013) and Slovakia (Demková et al., 2023). These concentrations seem very high, because they are focussed in thin organic topsoils, and they thus attracted intensive research. However, tillage would “dilute” that contaminated O horizon to ca. 30 cm and thus result in Pb concentration decrease to ca. one tenth, that would be comparable to average topsoil enrichment in the northern part of the Czech Republic (Fig. 7).

Coal extraction and combustion and twentieth century industrial development are among the most overestimated sources of LRTC by Pb in the Czech Republic (Adamec et al., 2024; Hošek et al., 2024), partly because of greatly overestimated Pb content in coal fly ash. Novák et al. (2003) calculated with 3000 mg kg^−1^ Pb in fly ash, while real concentration is ca. one-tenth (Czech et al., 2020; Matys Grygar et al., 2025). The Pb distribution in Czech agricultural soils (Fig. 1) shows no obvious spatial relation to major coal-fuelled power plants. Most local studies of LRTC impacts were conducted in forested mountainous areas, but many such areas in the Czech Republic and elsewhere in Europe hosted non-agricultural activities that produced Pb emissions since Mediaeval or even prehistoric times. Historical activities in ore regions are well documented (Bohdálková et al., 2018; von Scheffer et al., 2019, 2024), while abundant charcoal also supported emission-producing metallurgy and glassmaking (Kuneš et al., 2024; Tolksdorf et al., 2020).

A substantial share of anthropogenic Pb in peat archives from European forested areas originated in the pre-industrial period, as shown by dated contamination archives (Bindler, 2011; Bohdálková et al., 2018), and some prehistoric and historical sources remain unrecognised. Sampling in the Lužice Mountains near the boundary of DC and CL districts (Supplementary Information ESM2) revealed topsoil Pb enrichment of several hundred mg kg^−1^ (Fig.  7A, Fig. 6), with a spatial distribution indicating numerous local point sources. Heavy tails and high outliers in the nearby DC, CL and LI districts (Fig. 2) demonstrate scattered (spatially variable) contamination. The Jizera Mountains east of the Lužice Mountains, particularly the LI and JN districts, were also centres of glass production (Kuneš et al., 2024; Michczyński et al., 2022), including lead glass (Supplementary Information ESM2). Because glass production was largely overlooked by previous researchers (Ahado et al., 2021; Bednářová et al., 2016; Novák et al., 2003), soil contamination in the Jizera Mountains was attributed to coal combustion in the second half of the twentieth century. However, some local topsoils in the Lužice Mountains contain more Pb (Fig.  7 A) than the finest fly ash from a local Czech powerplant (100 mg kg^−1^, Matys Grygar et al., 2025) and fly ashes from most Polish coals (50–160 mg kg^−1^, Czech et al., 2020). In a peat core from the Jizera Mountains, Zuna et al. (2012) found substantial Pb deposition preceding the 20th-century maximum attributed to coal combustion (Novák et al., 2003), indicating an older Pb emission source. Michczyński et al. (2022) found a similar earlier peak on the Polish side of the mountain ridge and attributed it to local glassmaking, established in the late Middle Ages and peaking in the eighteenth and nineteenth centuries (Kuneš et al., 2024). Historical glassmaking also caused substantial Pb emissions elsewhere: at several glassworks in SE Sweden (Nyqvist et al., 2017), almost 140 t Pb was emitted in the 1970 s and nearly 100 t in the 1980 s (Alriksson et al., 2023).

A controversial and unresolved mechanism that may affect Pb in forest soil profiles is plant uptake of Pb, proposed by Reimann et al. (2008a, 2008b, 2008c) as a cause of elevated Pb in the forest floor. Fabian et al. (2017) retained natural processes as a contributor to topsoil Pb enrichment when evaluating diffuse contamination in Europe. Although critically discussed since formulation of the hypothesis on “plant pump for Pb” (Bindler, 2008, 2011; Shotyk, 2008; Steinnes, 2009), the issue remains unresolved as some arguments by Reimann et al. (2008a, 2008b) have not been disproved. Lead is not a biogenic element, and aboveground biomass of common boreal trees and plants contains only 0.2 to 1 mg kg^−1^ (Reimann et al., 2008a, 2008b, 2008c). However, wood ash from temperate and boreal trees contains 10 to 50 mg kg^−1^ Pb (Zając et al., 2018). Continuous biomass cycling in temperate and boreal forests through litter and trunk decomposition and recurrent fires has operated through much of the Holocene, i.e. for ca. 11 thousand years. This cycling could be further enhanced where woody part of forest biomass was used for centuries for charcoal and potash production for traditional glassmaking, as typical of mid-European mountain forests (Kuneš et al., 2024; Tolksdorf et al., 2020). Because Pb is strongly bound to organic matter (Bindler, 2011; Shotyk, 2008), cycling in the soil–plant system followed by biomass decay or burning could progressively concentrate Pb in organic topsoils. This effect may be stronger in peat cores because peat is the decomposition residue of large amounts of organic matter enriched in non-volatile elements. The emission loads calculated from soil profiles in mountain forests and peat bogs therefore provide upper estimates of diffuse anthropogenic contamination.

### Diffuse contamination of soils by Pb

Table 2 compares total Pb loads and topsoil increases derived from peat archives and soil analyses relevant to diffuse contamination in Central Europe, including results from this study. Topsoil enrichment of 3 mg kg^−1^ in the Klatovy Area (KT district), at least 50 km from the nearest known contamination source, corresponds to the upper range estimated for Europe by Fabian et al. (2017). Estimates for the Třebíč Area (TR district, Fig. S4.3 in ESM4) were based on the four profiles most remote from the local contamination centre (Fig. 9). The largest enrichment from remote sources occurred in the Novohradské Mountains (CK and CB districts), more than 40 km from the nearest known silver-smelting centre (Fig. S4.4 in ESM4). Enrichment increased from Klatovy through TR to the Novohradské Mountains (median altitudes 500, 600 and 750 m a.s.l., respectively), consistent with the altitudinal control of Pb emission load to soils discussed above and reported by Johanis et al. (2022).

**Table 2:** Overview of estimated diffuse contamination of topsoils in Europe by Pb. Topsoil increases calculated from total loads or vice versa are indicated by an arrow before the values in italics. The results of this work are listed as mean and Q1 and Q3 in parentheses

Geographic area	Total load (g m^−2^)	Topsoil increase (mg kg^−1^)	Reference
Global estimates	1 to 10	* → 2 to 20*	Shotyk et al. (2008); Steinnes (2009)
Scotland, soils	1 to 5	* → 2 to 10*	Farmer et al. (2016)
European agricultural topsoils	* → 0.5 to 1.5*	1 to 3	Fabian et al. (2017)
NE Poland, peat cores	0.7	* → 2*	Miszczak et al. (2020)
Norway, peat cores	2	* → 4*	Miszczak et al. (2020)
Austria, the Alps, peat core	2.7	* → 6*	von Scheffer et al. (2024)
Czech Republic, soils, Klatovy Area	* → 1.5*	3 (0; 5)	This work
Czech Republic, soils, Třebíč Area	* → 2*	4 (0; 12)	This work
Czech Republic, soils, the Novohradské Mts	* → 7*	16 (2; 42)	This work

Total loads inferred from peat archives early in this century (1 to 10 g m^−2^ globally, Table 2) imply topsoil enrichment of 10–20 mg kg^−1^ in agricultural soils ploughed to 30 cm. Such values occurred in the Novohradské Mountains and can be attributed to altitudinal control of LRTC. Because most European agricultural soils lie at lower altitudes, the lower estimate by Fabian et al. (2017) is more realistic. Peat-derived emission loads may apply mainly to mountainous areas and be positively biased by altitudinal control, historical activity and Pb cycling in the plant-soil system. Conversely, the estimate by Fabian et al. (2017) likely underestimates enrichment because altitudinal control makes the emission blanket spatially non-uniform (cannot be approximated by constant).

Environmental concern drove interest in Pb emission loads before the end of the twentieth century and contributed to the negative altitude of Europeans to the heavy industry. Farmer et al. (2016) attributed ca. half of Scottish topsoil Pb to LRTC emissions from the Industrial Revolution in southern United Kingdom since the eighteenth century. This represents substantial anthropogenic modification, reflecting Scotland’s low background, typical of Northern Europe (Reimann et al., 2012, 2018) and proximity to the origin of the European Industrial Revolution and its early environmental impacts. In most of Europe, diffuse contamination has contributed only weakly to local background (Reimann et al., 2012). In the Czech Republic, true diffuse contamination of up to 15 mg kg^−1^ would remain within natural background variability (Fig. 7B). In contrast, northern mountainous areas of the Czech Republic are more contaminated: here the topsoil enrichment reaches ca. 25 mg kg^−1^ in agricultural soils and 50 mg kg^−1^ in forest soils (Fig.  7A), with enrichment relative to subsoil accounting for 45 and 65% of topsoil Pb, respectively. Thus, the northern part of the Czech Republic has been affected by some local sources, not by regional diffuse contamination. The highest contamination reported here occurred in areas impacted by lead-glass production in the Lužice Mountains and farther east (Fig. 7 and Supplementary Information ESM2), yet even there the Pb concentration maxima remain below EU agricultural-soil limits of 80–300 mg kg^−1^ under regulation 86/278/EEC.

Another paradox in alarming Pb-emission assessments published around the turn of the century (Novák et al., 2003; Shotyk, 2008), which contributed to reduced European industrial activity, is overestimation of twentieth century industrial impacts on topsoil Pb. Current studies still repeat general warnings about “heavy metal emissions”, although modern technologies practically eliminate them, for example in coal-fired power generation (Adamec et al., 2024). Except for FM district, Czech topsoil Pb hotspots (Fig. 1 and S1.1 in Supplementary Information ESM1) resulted from activities predating twentieth century industrialisation (Supplementary Information ESM1). Scottish contamination reported by Farmer et al. (2016) also originated mainly during the early Industrial Revolution.

Czech topsoil contamination examined here (Table 2) is thus neither a toxicological nor a legislative concern, except possibly at localised hotspots around historical smelters and glassworks. Similarly, Tóth et al. (2016) considered risks from soil Pb contamination and consequent human intake negligible across Europe. Most of the existing contamination is historical.

### Limitations of this study

High-density soil monitoring subjected to exploratory statistical analysis represents the spatial distribution of cold HNO_3_-extractable Pb in ploughed horizons of agricultural soils (Fig. 1, ESM1). However, deeper horizons were not sampled in this monitoring because they are irrelevant to monitoring food production safety. Thus, the geogenic portion of Pb could not be directly recognised and required complementary sampling. Moreover, national monitoring provides no spatially representative forest-soil dataset, resulting in poor coverage of mountainous and usually forested areas, including sites close to historical contamination sources typical of non-agricultural landscapes, such as the Lužice Mountains with their historical glassmaking (ESM2). Complementary sampling of forested areas had poorer spatial coverage and may have left undocumented contamination sources unrecognised. Sampling forested soils was thus indispensable and revealed larger LRTC in “soils under tree crowns”.

## Conclusion

Although soil contamination by Pb is no longer considered an urgent problem of European soil studies, the interest in that topic has left a considerable trace in research literature, extensive datasets of soil contamination data, and several persistent yet unsolved tasks related to processing geochemical soil maps and critical evaluation of anthropogenic impacts on soils. Additionally, soil contamination is relevant in newly industrialised areas of Asia and South America, where the above presented approaches can be implemented to improve the reliability of their conclusions. This work demonstrates the use of exploratory functional data analysis and geospatial tools to characterise point and scattered contamination and its altitudinal control from the national soil monitoring programme, that can be recommended for use in all suitable datasets. Preceding studies on soil contamination by Pb have overestimated diffuse contamination from remote sources in the twentieth century and underestimated local historical sources, usually undocumented, such as decorative glass production in the north of the Czech Republic. This work also demonstrates possible biases of the preceding evaluation of true diffuse soil contamination by Pb and the indispensability of sampling of soil profiles to characterise it. Forest soils in mountain areas provide upper, overestimated values of diffuse contamination, while lowland agricultural soils receive less diffuse contamination.

## Supplementary Information

Below is the link to the electronic supplementary material.

Supplementary Material File 1 (PDF 2.33 MB)

Supplementary Material File 2 (PDF 1.04 MB)

Supplementary Material File 3 (XLSX 65.1 KB)

Supplementary Material File 4 (PDF 1.00 MB)

Supplementary Material File 5 (PDF 503 KB)

## Data Availability

The RKP datasets cannot be freely provided. The results of soil profile analyses are available on request from the corresponding author.
