# Supplementary material for: Revisiting estimates of diffuse contamination of agricultural soils by Pb
Source: Environ Monit Assess. 2026 Aug 5;198(9):916. doi: 10.1007/s10661-026-15684-5 (PMC13442501; doi:10.1007/s10661-026-15684-5)
Supplement: Supplementary file 1 [file 10661_2026_15684_MOESM1_ESM.pdf]

Major Pb contamination sources in the Czech Republic

T. Matys Grygar[1], Š. Tůmová[1,2]

[1] Institute of Inorganic Chemistry, Czech Academy of Sciences, 250 68 Řež, Czech Republic

[2] Faculty of Environment, J. E. Purkyně University in Ústí nad Labem, Pasteurova 3632/15, 400 96 Ústí nad Labem, Czech Republic

Supplementary Material SM1 to the paper "Revisiting estimates of diffuse contamination of agricultural soils by Pb" by T. Matys Grygar et al.

Interpreted soil contamination map

Fig. S1.1 is a simplified map of the Pb contamination map in the Czech Republic with a numbered list of the major areas; their association with contamination sources is listed in Table S1.1. The interpretation is based on preceding systematic research of soil monitoring in the Czech Republic (Vácha et al. 2015; Bednářová et al. 2016; Matys Grygar et al. 2023; Skála et al. 2024).

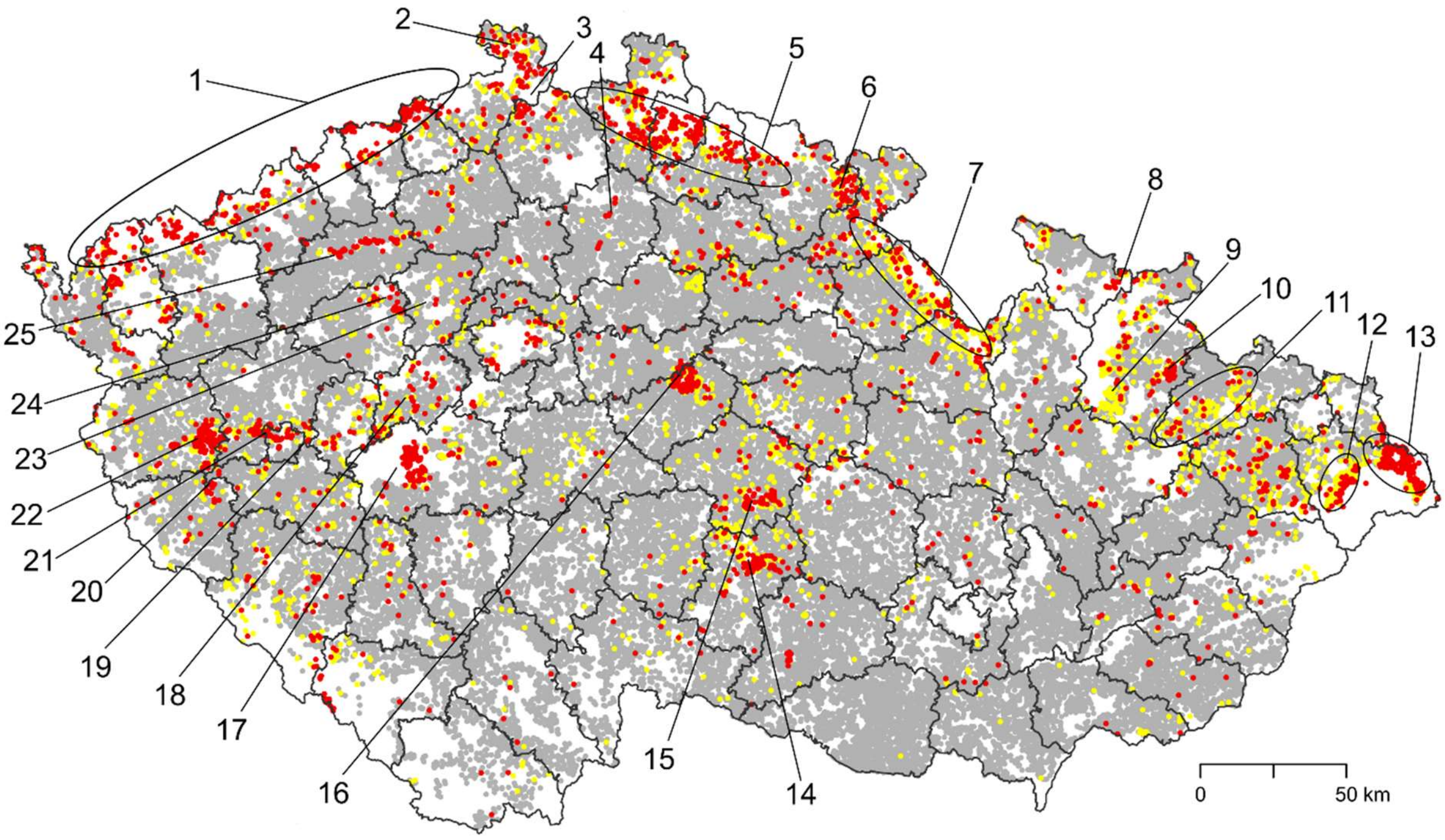

Figure S1.1. Interpreted map of Pb contamination in the Czech agricultural soils obtained from monitoring using cold 2 M HNO_3 extraction. Numbers are explained in Table S1.1. The legend to the figure is present in the paper as Fig. 1.

Soil contamination sources

Metallurgy belongs to the major sources of soil contamination in the Czech Republic. The efficiency of early ore processing and smelting has very slowly improved over centuries, and thus early smelting

was spatially limited, but its impacts have been considerable. Cupellation, i.e., the use of Pb separation of Ag from ores and its refining, used since pre-historic times to early modern times, has produced considerable European contamination by Pb from numerous local ore regions, initially mainly in southern Europe (Erel et al. 2021) but later mainly in Central Europe (Longmann et al. 2020; Novák et al. 2003; Matys Grygar et al. 2023). Central European mountain ore regions contributed significantly since Mediaeval times (Bohdálková et al. 2018; Longman et al. 2020; Hošek et al. 2024). Regional character of the contamination in Central Europe (Longmann et al. 2020) resulted from numerous local centres, including the area of the Czech Republic (Table S1.1). In the 18th and 19th centuries, small local smelters in the Czech Republic were stepwise closed, and only one major metallurgy enterprise remained in operation in Příbram, which kept production of non-ferrous metals from ores and later switched to recycling Pb accumulators (Ettler et al. 2006).

Table S1.1. Assignment of major soil Pb hotspots to contamination sources

Source	Shape	Contamination sources/reasons
Ag or Ag+Pb metallurgy	Almost isometric (circular)	Stříbro (22), Příbram (17), Kutná Hora (16), Havlíčkův Brod (15), Jihlava (14), Horní Benešov (10)
Polymetallic ores including Au and Cu		Zlaté Hory (8)
Fe metallurgy	Almost isometric (circular)	Rýmařov (9), Frýdlant nad Ostravicí (12), Králův Dvůr (18), Plzeň (19), Heřmanova Huť (21)
Fe and steel metallurgy		Ostrava+Třinec (13), Plzeň (19), Kladno (23)
Glassmaking	Almost isometric	Lužice Mountains (3)
Glassmaking	Elongated	Jablonec nad Nisou area (5) – “Crystal valley”
Geology+metallurgy	Elongated	Ore Mountains (1)
Geology	Elongated	Flysch (11)
Uncertain	Anisometric	Šluknov Area (2)
Coal-based industry	Elongated	Svatoňovice Area (6), NE part of RA district (24)
LRTC from unclear sources	Elongated	Orlice Mountains (7) – orographic barrier
Floodplains	Linear	The Jizera River (4), the Ohře River (25)

Iron- and steel production have contributed significantly to Pb emissions in the Czech Republic (Matysek et al. 2008; Matys Grygar et al. 2023; Bílková et al. 2026), because iron ore sintering, coal use, iron smelting, and steel production are related to Pb emissions (Lau et al. 2021). Coal combustion produces fly ash, in whose finest fractions Pb is enriched to ca 100 mg kg^{-1} (Ratafia-Brown, 1994; Lanzerstorfer, 2018; Czech et al., 2020). Major metallurgy centres and contamination associated with coal combustion are listed in Table S1.1.

Less known but locally relevant was the former use of lead compounds for the production of glass, either with a small addition of Pb to improve glass melting and stabilise glass against damage by light (solarisation) or by adding PbO in amounts of up to ca. 1/3 weight of the glass to improve its

refraction and processability to final decorative goods and custom jewellery. In Czechia, one case study documented Pb contamination around former glassworks in Světlá nad Sázavou (Knésl et al. 2010). The lead emissions from glassmaking have been very rarely documented in the literature. The possible importance of glassmaking can be documented by the case of several glassworks in southern Sweden, where the Pb pollution peaked in the 1970s by almost 140 t Pb emitted to the atmosphere, followed by a bit less than 100 t Pb in the 1980s (Alriksson et al. 2023). Then the production of lead glass decreased roughly coevally with the phase-out of leaded gasoline use and massive use and improvements of electrostatic precipitators in coal-fired power plants. Interestingly, the largest concentrations of Pb in south Norway soils were found along the south to southeast coast of southern Norway (Steinnes and Njåstadt 1995; Steinnes 2009; Steinnes and Lierhagen 2018), and most contaminated dust deposits in southern Norway in 1985 originated from east to southwest (Steinnes 2009). Environmental scientists did not pay attention to this burden in the early 21st century, similarly as Czech researchers who studied Pb in soils of the Czech Republic overlooked the local glassmaking impacts in north Czechia. The actual impact of glass production on Pb contamination in soils in the Czech Republic is documented by the case of the Lužice Mountains in Supplementary Material 2.

Hotspots of soil Pb contamination and the most relevant Pb sources in the Czech Republic

Fig. S1.2 shows major contamination hotspots obtained by Getis-Ord Gi* for 80 neighbours by ArcGIS (Esri, USA). The hotspots are assigned to contamination sources in Table S1.1. The geospatially smoothed pattern in that figure extracts the major contamination sources and patterns in the Czech Republic. Production of Ag and Pb in Medieval and early modern times belonged among the most important Pb contamination sources in the Czech Republic (Novák et al. 2003; Matys Grygar et al. 2023) and Central and southern Europe (Longmann et al. 2020). Historical and industrial iron- and steelmaking have also been relevant (Table S1.1). The almost isometric shape of many hotspots is caused by a combination of localised contamination from point sources and smoothing caused by Gi* algorithm. Besides that, there are fewer isometric (areal) hotspots usually at slopes of mountain ridges. Soil Pb in the Ore Mountains is elevated due to a combination of historical metallurgy and elevated local background (Bohdálková et al. 2018; Hošek et al. 2024). The contamination of the Czech soils, in particular in mountain areas, has mainly been attributed to coal-based heavy industry and energy production in the 20th century (Novák et al. 2003). However, many areas have received the majority of contamination earlier, as it has already been proven that the Ore Mountains along the Czech-German border have received more contamination from local metallurgy (Tarvainen et al. 2020), mainly in the 16th century (Bohdálková et al. 2018; Adamec et al. 2024; Hošek et al. 2024). Generally, coal combustion played a much smaller role in Pb emissions than it has been declared by national researchers since the 1990s. E.g. Novák et al. (2003) based their calculation of LRTC from coal-fuelled power plants on an estimate that fly ash contains 3000 mg kg^{-1} Pb. That estimate was

overestimated at least by one order of magnitude, if it is compared to the real content of Pb in the finest fly ashes, which contain most Pb (Adamec et al. 2024).

Large areas show less localised (more scattered) associations of elevated and high values, e.g. along the mountain ridges along the Czech-Polish state boundary (areas show part of heavy tails of scattered contamination; areas 5 to 7 in Fig. S1.1). Anthropogenic contamination in O and less in A horizons of soils in those boundary mountains has been reported in many studies (Karczewska and Kabała 2002; Sucharová et al. 2011; Szopka et al. 2013; Kváčová et al. 2015; Bińczycki et al. 2020). The shape of the corresponding hotspots is dictated by terrain topography – the contaminated agricultural soils are mostly on the mountain slopes exposed to the westerly winds.

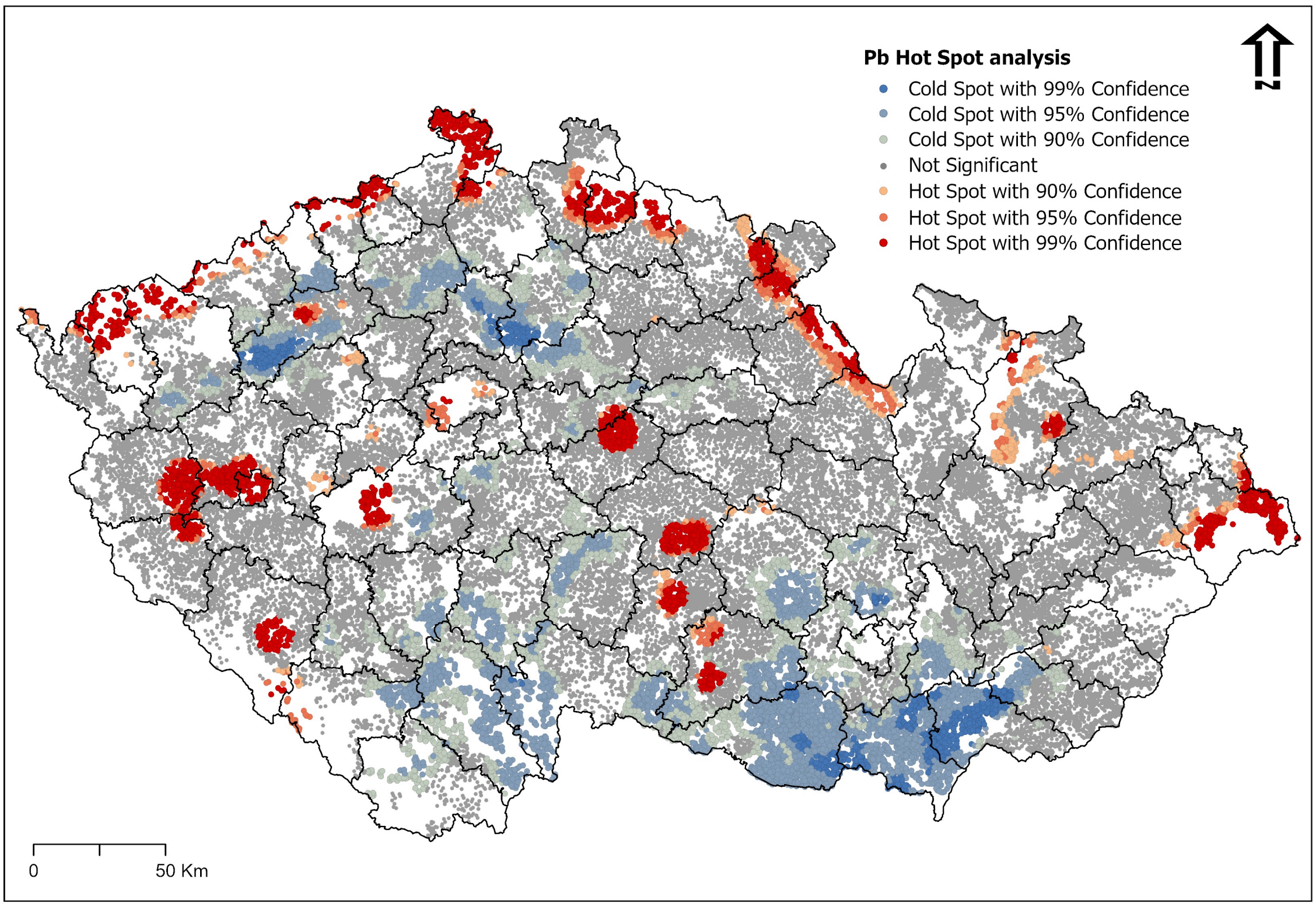

Figure S1.2. Hotspots of Pb contamination in agricultural soils of the Czech Republic obtained by geostatistical analysis. It shows a highly smoothed simplification of the map in Fig. S1.1.

Contamination of floodplains

Contamination is abundant in the soils of floodplains of several Czech rivers draining ore regions, the surroundings of historical smelters, and flowing through big cities and local industrial enterprises from the beginning of industrialisation (Kylich 2021; Matys Grygar et al. 2023). Floodplain hotspots are better visible in the simple map with a classified layer (Fig. S1.1) as narrow strips around many lowland rivers, formerly free-flowing in broad and flat alluvial plains (Matys Grygar et al. 2013, 2016; Nováková et al. 2013, 2016; Faměra et al. 2021). In Fig. S1.1, area 4 is the floodplain of the Jizera

River (Matys Grygar et al. 2013), and area 25 is the floodplain of the Ohře River (Matys Grygar et al. 2016). Contamination in those river stretches frequently originated many tens km far upstream. Because of their anisometric shape, floodplain hotspots are underrepresented in Getis-Order Gi* hotspots (Fig. S1.2). In a single place in the Ohře River floodplain, there is a sufficient number of samples to produce a hotspot.

Interesting is also the distribution of coldspots in Fig. S1.1. Remarkably low Pb are found in lowlands, where soils are derived from sedimentary rocks. Particularly common are low-Pb areas in the southern part of the Czech Republic. Forest humus in the southern part of CR is thus less contaminated by Pb than the northern part, in particular than the most contaminated strip along the Czech-Polish state boundary (Sucharová et al. 2011).

References

Adamec S, Tůmová Š, Hošek M, Lučić M, Matys Grygar T (2024) Pitfalls of distinguishing anthropogenic and geogenic reasons for risk elements in soils around coal-fired power plants: from a case study in the Northwestern Czech Republic to general recommendations. J Soils Sediments 24, 1274–1288. DOI:10.1007/s11368-024-03726-9

Bednářová Z, Kalina J, Hájek O, Sáňka M, Komprdová K (2016) Spatial distribution and risk assessment of metals in agricultural soils. Geoderma 284, 113–121

Bílková G, Matys Grygar T, Elznicová J, Hýlová V, Lučić M (2026) Geographic control of emissions from iron- and steelmaking in Třinec, Czech Republic caught by the Beskid slopes. Carpathian Journal of Earth and Environmental Sciences, 21, 171 – 188. DOI:10.26471/cjees/2026/021/361

Bińczycki T, Weber J, Mielnik L, Asensio C (2020) Lead isotope ratios in Podzol profiles as a tracer of pollution source in the subalpine zone of the Karkonosze National Park, Sudety Mts (south-western Poland). Catena 189, 104476. DOI:10.1016/j.catena.2020.104476

Bohdálková, L., Bohdálek, P., Břízová, E., Pacherová, P., Kuběna, A.A. (2018) Atmospheric metal pollution records in the Kovářská Bog (Czech Republic) as an indicator of anthropogenic activities over the last three millennia. Science of the Total Environment 633, 857–874

Czech T, Marchewicz A, Sobczyk AT, Krupa A, Jaworek A, Śliwiński Ł, Rosiak D (2020) Heavy metals partitioning in fly ashes between various stages ofelectrostatic precipitator after combustion of different types of coal. Process Safety and Environmental Protection 133, 18–31

Erel Y, Pinhasi R, Coppa A, Ticher A, Tirosh O, Carmel L (2021) Lead in Archeological Human Bones Reflecting Historical Changes in Lead Production. Environ. Sci. Technol. 55, 14407−14413.

Ettler V, MihaljevičM, Šebek O, Molek M, Grygar T, Zeman D (2006) Geochemical and Pb isotopic evidence for sources and dispersal of metal contamination in stream sediments from the mining and smelting district of Příbram, Czech Republic. Environmental Pollution 142, 409-417

Faměra M, Matys Grygar T, Ciszewski D, Czajka A, Álvarez-Vázquez MÁ, Hron K, Fačevicová K, Hýlová V, Tůmová Š, Světlík I, Zimová K, Dvořáková K, Szypula B, Hošek M, Henych J (2021) Anthropogenic records in a fluvial depositional system: The Odra River along The Czech-Polish border Anthropocene 34. DOI:10.1016/j.ancene.2021.100286

Hošek M, Pavlíková P, Šoltýs M, Tůmová Š, Matys Grygar T (2024) Distinguishing Geogenic Load and Anthropogenic Contribution to Soil Contamination in Mineralised Mountain Landscape of Ore Mountains (Czech Republic) Using Cumulative Distribution Functions. Land 13, 218. DOI:10.3390/land13020218

Karczewska A, Kabala C (2002) Pierwiastki śladowe v glebach Parku narodowego Gór Stołowych (Trace elements in soils in the Stołowe Mountains National Park). Szczeliniec 6, 133-160. https://www.researchgate.net/publication/309761902

Knésl I, Kříbek B, Mihaljevič M, Šikl J, Buda J, Lukeš P (2010) Kontaminace půd těžkými kovy v okolí závodu na výrobu olovnatého skla ve Světlé nad Sázavou (Soil contamination by heavy metals around lead glass production in Světlá nad Sázavou). In Geoscience Research Reports for 2009, 223-228. Czech Geological Survey, Prague

Kváčová M, Ash C, Borůvka L, Pavlů L, Nikodem A, Němeček K, Tejnecký V, Drábek O (2015) Contents of Potentially Toxic Elements in Forest Soils of the Jizera Mountains Region. Environ Model Assess 20:183–195. DOI:10.1007/s10666-014-9425-3

Kylich T (2021) Vytvoření a vysvětlení mapy obsahů Cd a Cu v půdách ČR s důrazem na říční krajinu (Creation and explanation of maps for soil Cd and Zn in Czech Republic with stress on fluvial landscapes). Diploma These, Faculty of Environment, J. E. Purkyně Universty in Ústí nad Labem, Czech Republic

Lanzerstorfer C (2018) Fly ash from coal combustion: Dependence of the concentration of various elements on the particle size. Fuel 228, 263–271

Lau LL, Strezov V, Gonçalves MVB, Bagatini MC (2021). Trace elements emission in iron ore sintering: A review. Environmental Advances 6, 100123. DOI:10.1016/j.envadv.2021.100123

Longman J, Ersek V, Veres D (2020) High variability between regional histories of long-term atmospheric Pb pollution. Scientific Reports 10:20890. DOI:10.1038/s41598-020-77773-w

Matys Grygar T, Nováková T, Bábek O, Elznicová J, Vadinvá N (2013) Robust assessment of moderate heavy metal contamination levels in floodplain sediments: A case study on the Jizera River, Czech Republic. Science of the Total Environment 452, 233-245

Matys Grygar T, Elznicová J, Kiss T, Smith HG (2016) Using sedimentary archives to reconstruct pollution history and sediment provenance: The Ohre River, Czech Republic. Catena 144, 109-129

Matys Grygar T, Elznicová J, Tůmová Š, Kylich T, Skála J, Hron K, Álvarez-Vázquez MÁ (2023) Moving from geochemical to contamination maps using incomplete chemical information from long-term high-density monitoring of Czech agricultural soils. Environmental Earth Sciences 82:6. DOI:10.1007/s12665-022-10692-3

Matysek D, Raclavska H, Raclavsky K (2008) Correlation Between Magnetic Susceptibility and Heavy Metal Concentrations in Forest Soils of the Eastern Czech Republic. Journal of Environmental and Engineering Geophysics 13, 13-26

Novák M, Emmanuel S, Vile MA, Erel Y, Véron A, Pačes T, Wieder RK, Vaněček M, Štěpánová M, Břízová E, Hovorka J (2003) Origin of lead in eight central European peat bogs determined from isotope ratios, strengths, and operation times of regional pollution sources. Environmental Science & Technology 37, 437-445. DOI:10.1021/es0200387

Nováková T, Matys Grygar T, Bábek O, Faměra M, Mihaljevič M, Strnad L (2013). Distinguishing regional and local sources of pollution by trace metals and magnetic particles in fluvial sediments of the Morava River, Czech Republic. Journal of Soils and Sediments 13, 460-473

Nováková T, Matys Grygar T, Kotková K, Elznicová J, Strnad L, Mihaljevič M (2016) Pollution assessment using local enrichment factors: the Berounka River (Czech Republic). Journal of Soils and Sediments 16, 1081-1092

Ratafia-Brown JA (1994) Overview of trace element partitioning in flames and furnaces of utility coal-fired boilers. Fuel Processing Technology 39, 139-157

Skála J, Matys Grygar T, Achasova A (2024) Novel definition of local baseline values for potentially toxic elements in Czech farmland using adaptive spatial weighting. Applied Geochemistry 170, 106082. https://doi.org/10.1016/j.apgeochem.2024.106082

Steinnes E (2009) Comment on "Geochemical gradients in soil O-horizon samples from southern Norway: Natural or anthropogenic?" by C. Reimann, P. Englmaier, B. Flem, L. Gough, P. Lamothe, Ø. Nordgulen, and D. Smith". Applied Geochemistry 24, 2019–2022

Steinnes E, Lierhagen S (2018) Geographical distribution of trace elements in natural surface soils: Atmospheric influence from natural and anthropogenic sources. Applied Geochemistry 88, 2-9

Steinnes E, Njåstadt O (1995) Enrichment of metals in the organic surface layer of natural soil: Identification of contributions from different sources. The Analyst 120(5), 1479-1483. DOI: 10.1039/an9952001479

Sucharová J, Suchara I, Holá M, Reimann C, Boyd R, Filzmoser P, Englmaier P (2011) Linking chemical elements in forest floor humus (Oh-horizon) in the Czech Republic to contamination sources. Environmental Pollution 159, 1205-1214

Szopka K, Karczewska A, Jezierski P, Kabala C (2013) Spatial distribution of lead in the surface layers of mountain forest soils, an example from the Karkonosze National Park, Poland. Geoderma 192, 259-268. DOI:10.1016/j.geoderma.2012.08.022

Tarvainen T, Reichel S, Müller I, Jordan I, Hube D, Eurola M, Loukola-Ruskeeniemi K (2020) Arsenic in agro-ecosystems under anthropogenic pressure in Germany and France compared to a geogenic As region in Finland. Journal of Geochemical Exploration 217, 106606

Skála J, Matys Grygar T, Achasova A (2024) Novel definition of local baseline values for potentially toxic elements in Czech farmland using adaptive spatial weighting. Applied Geochemistry 170, 106082. DOI:10.1016/j.apgeochem.2024.106082

Vácha R, Skála J, Čechmánková J, Horváthová V, Hladík J (2015) Toxic elements and persistent organic pollutants derived from industrial emissions in agricultural soils of the Northern Czech Republic. J Soils Sediments 15, 1813–1824. DOI:10.1007/s11368-015-1120-8
