# Supplementary material for: Revisiting estimates of diffuse contamination of agricultural soils by Pb
Source: Environ Monit Assess. 2026 Aug 5;198(9):916. doi: 10.1007/s10661-026-15684-5 (PMC13442501; doi:10.1007/s10661-026-15684-5)
Supplement: Supplementary file 2 [file 10661_2026_15684_MOESM2_ESM.pdf]

Soil contamination in the Lužice Mountains caused by glass production

Š. Tůmová[1,2], L. Kuglerová[2], T. Matys Grygar[1]

[1] Institute of Inorganic Chemistry, Czech Academy of Sciences, 250 68 Řež, Czech Republic

[2] Faculty of Environment, J. E. Purkyně University in Ústí nad Labem, Pasteurova 3632/15, 400 96 Ústí nad Labem, Czech Republic

Supplementary Material SM2 to the paper "Revisiting estimates of diffuse contamination of agricultural soils by Pb" by T. Matys Grygar et al.

Introduction to the study area

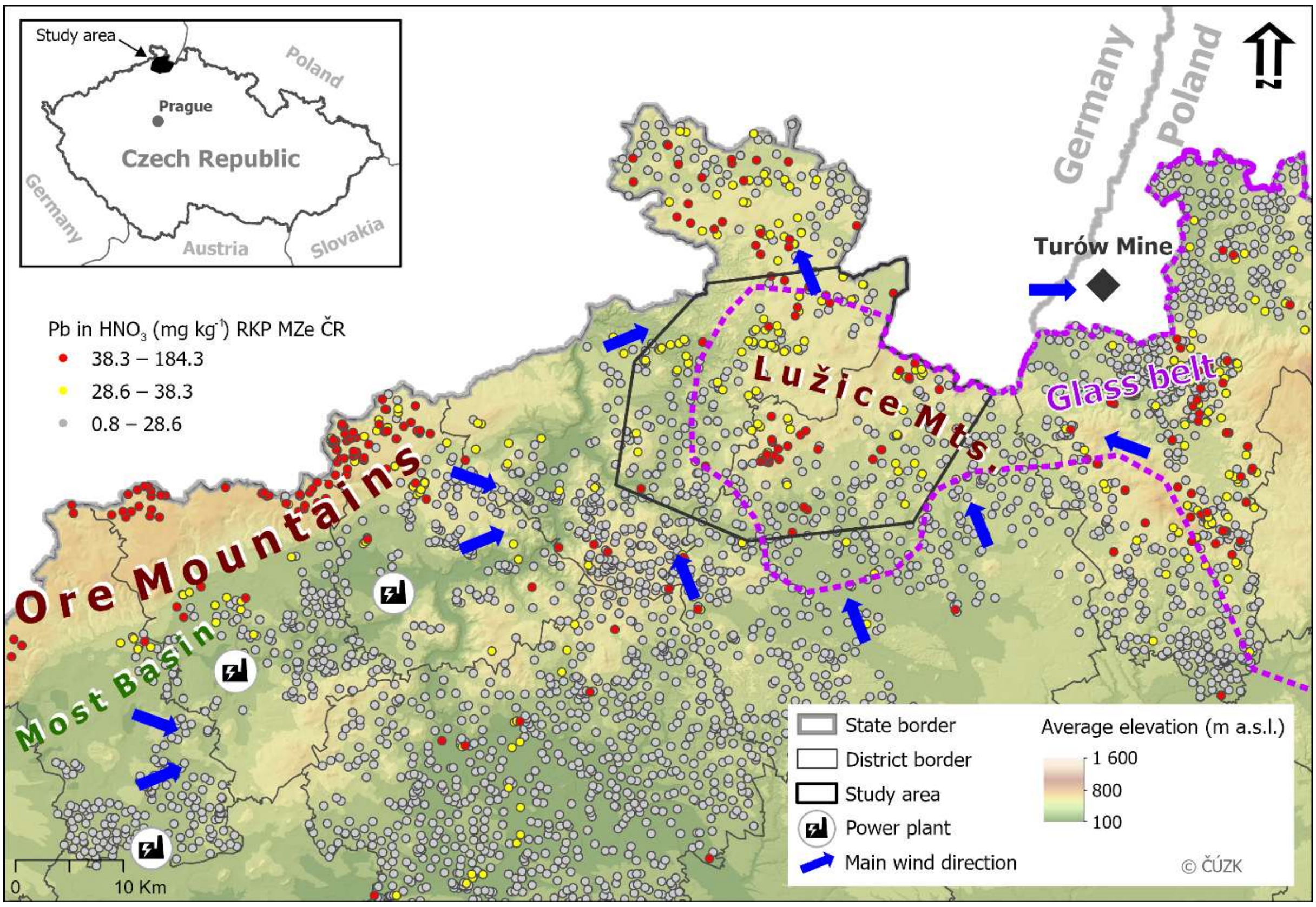

Figure S2.1. Map of the north-west part of the Czech Republic with the Lužice Mountains and its surroundings. Circles show sites of the national agricultural soil monitoring, with red circles indicating considerable contamination by acid-extractable Pb. Noteworthy is the lack of agricultural soil monitoring in the elevated areas inside the Lužice Mountains, where we thus performed soil sampling and analysis.

The study area of the Lužice Mountains (Fig. S2.1) is a part of a broader geographic area formerly known as the "Black Triangle", encompassing the borders of the Czech Republic, Germany, and Poland (Blažková 1996). During the second half of the 20th century, the Black Triangle was characterised by extreme forest damage by emissions of SOx and NOx from heavy industry and energy production based

on the soft coal mining and utilisation (Blažková 1996; Kuneš et al. 2024). One of the possible emission sources could also be the Polish Turów energy complex utilising lignite from the Turów coal mine (Fig. S2.1). Preceding authors attributed soil contamination by Pb in the Black Triangle to coal combustion (Novák et al. 2003; Zuna et al. 2011). Our recent studies (Adamec et al. 2024; Matys Grygar et al. 2026), however, showed that the soil Pb contamination in the Czech Republic around the Most Basin power plants has not been related to the coal combustion.

Due to the prevailing westerly and south-westerly wind patterns, the monitored area in the Lužice Mountains could have been exposed to potential long-range transported contamination (LRTC) from other major Central European centres, such as the coal basins of the Most Basin to the west (power plants south-east of the Ore Mountains in Fig. S2.1). The Turów energy complex is less likely to contribute to the contamination of the Lužice Mountains because the wind direction is unfavourable for this source (Fig. S2.1). Ridges and peaks in the Lužice Mountains act as a natural orographic barrier capable of intercepting LRTC. A significant portion of the Lužice Mountains is covered by forest stands not included in the state monitoring by the Ministry of Agriculture of the Czech Republic (RKP MZe ČR) ("blind spots" – areas without samples in Fig. S2.1).

History and technology of glass production in the Lužice Mountains

Historical production of decorative glass is a lesser-known but relevant contamination source in the Lužice Mountains (Čiháková 2024; Kuglerová 2025). The Lužice Mountains are in the western part of a region called "glass belt" (Fig. S2.3 and S2.4) with a unique, continuous glassmaking tradition dating back to the 13th century (Drahotová 2005; Kuneš et al. 2024). The development of this industry was facilitated by an abundance of natural resources for glassmaking in the Lužice Mountains, particularly fuelwood and quartz sands, while the mountainous terrain and sandy soils did not provide suitable conditions for agriculture. Historical glass-melting technologies can have considerable environmental importance. To achieve clarity, high lustre, and the removal of gas bubbles (a process known as refining or fining), lead oxide (PbO) and arsenic compounds were added to the glass batch (Drahotová et al. 2005). While silica sand melts at very high temperatures, lead and arsenic oxides begin to volatilize under these conditions. Consequently, the emissions of the relative volatile As and Pb oxides were emitted into the surrounding landscape.

During the second half of the 17th century, the production of transparent glass, characterised by its exceptional clarity, brilliance, and hardness, containing considerable portions of PbO, so-called lead glass, commenced in the Bohemian lands (Drahotová et al. 2005). Lead glass, particularly suitable for engraving and cutting, became known as Bohemian crystal.

Strategy for addressing data gaps in the national soil monitoring

State monitoring of agricultural soils, included in RKP, is primarily oriented towards crop fields and grasslands. In the rugged and forested terrain of the Lužice Mountains, this focus has resulted in

extensive "blind spots", unmapped areas, where no soil sampling has been performed (Fig. S2.1). As documented in the map (Fig. S2.2), our research was focused on filling these gaps. To obtain good spatial coverage, we sampled topsoil 20 cm using a soil auger and analysed samples for total Pb content using XRF. The results are shown hereafter.

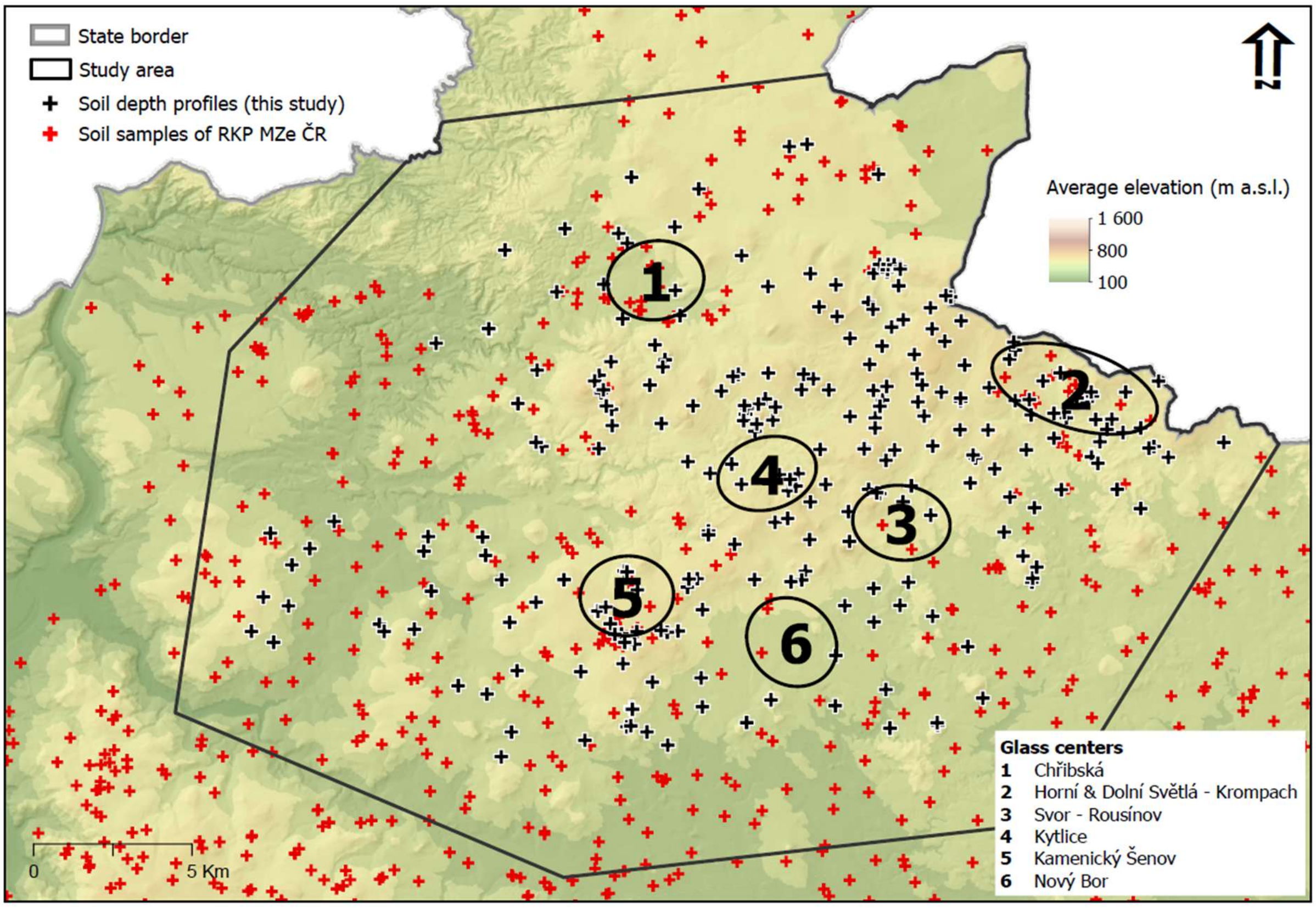

Figure S2.2. Our sampling (black crosses) filled the “blind spots” in state agricultural soil monitoring (red crosses). Numbered are known historical glass centres (Čiháková 2024).

Utilising historical sources and GIS tools, the documented historical glassmaking sites (glassworks, grinding mills, and mirror factories) were identified in the study area (Čiháková et al. 2024, Kuglerová 2025; see glass centres in Fig. S2.2). In their vicinity, more than 260 topsoil samples were acquired, and sometimes soil depth profiles were also obtained, reaching depths of 80 to 110 cm. The position of all sampled sites is shown in Fig. S2.2 (black crosses).

Interpretation of spatial distribution and the origin of contamination in the Lužice Mountains

The spatial distribution of lead (Pb) in the soils of the Lužice Mountains, as detailed in the proportional symbol map of concentrations (Fig. S2.3), provides critical evidence for the existence of point pollution sources in the mountain valleys. The map reveals that the highest total Pb concentrations (red points within the interval of 132–785 mg/kg) are concentrated in the central part of the study mountains, particularly on the ridges and peaks. This mapping framework allows for the exclusion of long-range

transport from major industrial centres, such as the Most Basin to the west or the Polish Turów mine and power plant to the north, as the dominant source of contamination. As documented by the comparative map in a broader context (Fig. S2.1), Pb concentrations in soils around the Lužice Mountains decrease toward these distant sources, indicating that the Lužice Mountains form an isolated area with high internal heterogeneity of soil contamination by Pb (Fig. S2.3) centred around the glassmaking centres (Fig. S2.2). Had the pollution originated from regional deposition from these basins, the peripheral parts of the mountain range would exhibit an increasing gradient toward them; however, the opposite contamination pattern was obtained (Fig. S2.3).

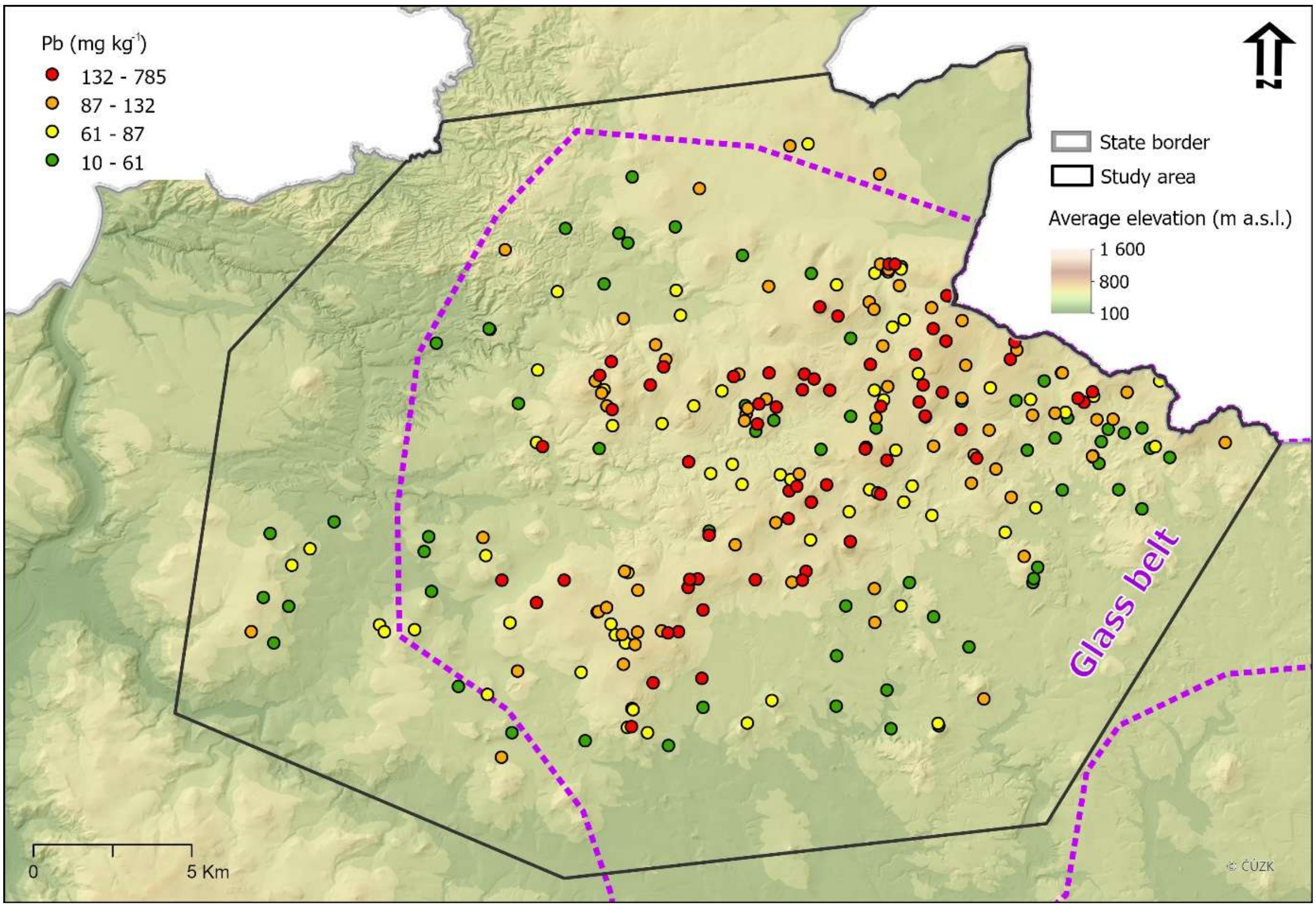

Figure S2.3. Total Pb concentrations in the topsoil 20 cm obtained by our sampling also cover forested areas of the Lužice Mountains.

The spatial distribution of Pb in the Lužice Mountains (Fig. 2.3) is very heterogeneous. Instead of the diffuse, uniform pollution that could be expected for LRTC from remote sources, the soils in the Lužice Mountains show significant hotspots of contamination. The Getis-Ord Gi* analysis identified clusters of high-concentration points reaching 90 % to 99 % confidence levels (red circles in Fig. S2.4). These hotspots are primarily located in the central and eastern parts of the range and show a strong correlation with local morphology and historical glassmaking centres. Local mountain ridges and peaks are known to act as natural orographic barriers intercepting emissions and thus capturing them rather close to the sources (Bílková et al. 2026). The high heterogeneity shown in the Fig. S2.4 map (the

alternation of heavily contaminated points with those marked as "not significant") is a crucial finding supporting the local origin of the pollution. If coal combustion in the Most Basin or the Turów energy complex plant were the dominant sources, the map would present a much more homogeneous pattern with gradual gradients rather than isolated and sharply delineated hotspots.

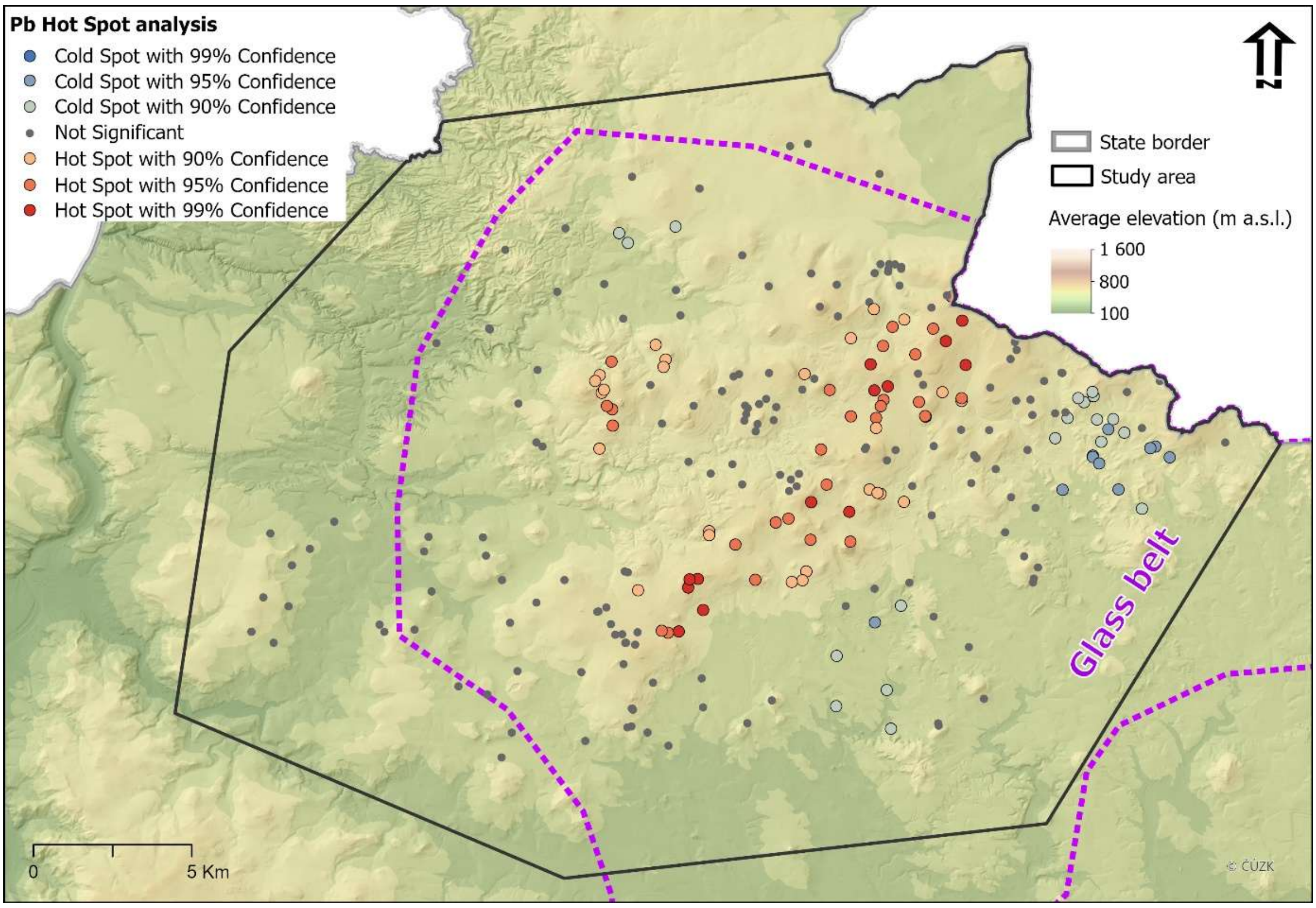

Figure 2.4. Hot spot/cold spot analysis of total Pb in top 20 cm of soils in the Lužice Mountains obtained by Getis-Ord Gi* spatial analysis.

References

Adamec S, Tůmová Š, Hošek M, Lučić M, Matys Grygar T (2024) Pitfalls of distinguishing anthropogenic and geogenic reasons for risk elements in soils around coal-fired power plants: from a case study in the Northwestern Czech Republic to general recommendations. J Soils Sediments 24, 1274–1288. DOI:10.1007/s11368-024-03726-9

Bílková G, Matys Grygar T, Elznicová J, Hýlová V, Lučić M (2026) Geographic control of emissions from iron- and steelmaking in Třinec, Czech Republic caught by the Beskid slopes. Carpathian Journal of Earth and Environmental Sciences, 21, 171 – 188. DOI:10.26471/cjees/2026/021/361

Blažková M (1996) Black Triangle - the most polluted part of Central Europe. In: P. E. Rijtema and V. Eliáš (eds.), Regional Approaches to Water Pollution in the Environment, 227-249. Kluwer Academic Publishers. DOI:10.1007/978-94-009-0345-6_11

Čiháková N. (2024) Mapování kontaminace půd spojené se sklářskou tradicí v Lužických horách (Mapping of Soil Contamination Related to Glass Tradition in the Lužice Mountains). Bachelor Theses, Faculty of Environment, J. E. Purkyně University in Ústí nad Labem, Czech Republic

Drahotová O. (2005) Historie sklářské výroby v českých zemích. Díl 1. Od počátků do konce 19. století (History of Glassmaking in Czech Lands, Part 1: From Beginning to the end of 19th century). Czech Glass Society and Academia, Prague, 759 pages. ISBN 80-200-1287-7

Kuglerová (2025) Posouzení biodostupnosti kontaminace půd ve východní části Lužických hor prvky As, Cd, Pb a Zn (Evaluation of soil contamination bioavailability in eastern part of the Lužice Mountains by As, Cd, Pb, and Zn). Bachelor Theses, Faculty of Environment, J. E. Purkyně University in Ústí nad Labem, Czech Republic

Kuneš I, Podrázský V, Baláš M, Remeš J, Navrátil P, Kuc M, Smejkal J, Bursíková J, Paluch JG (2024) The history of the Jizera Mts forests in the former heavily polluted area of Central Europe. The Anthropocene Review 11(2), 463–487

Matys Grygar T, Adamec S, Tůmová Š, Burdová H, Kříženecká S, Bretschneiderová D, Lučić M, Pavlů I (2026) Identification of coal combustion impacts on soil contamination by risk elements needs empirical holistic approach: case study in the Most Basin, Czech Republic. Environmental Earth Sciences 84, 16. DOI:10.1007/s12665-025-12458-z

Novák M, Emmanuel S, Vile MA, Erel Y, Véron A, Pačes T, Wieder RK, Vaněček M, Štěpánová M, Břízová E, Hovorka J (2003) Origin of lead in eight central European peat bogs determined from isotope ratios, strengths, and operation times of regional pollution sources. Environmental Science & Technology 37, 437-445. DOI10.1021/es0200387

Zuna M, Mihaljevič M, Šebek O, Ettler V, Handley M, Navrátil T, Goliáš V (2011) Recent lead deposition trends in the Czech Republic as recorded by peat bogs and tree rings. Atmospheric Environment 45, 4950-4958
