# Supplementary material for: Revisiting estimates of diffuse contamination of agricultural soils by Pb
Source: Environ Monit Assess. 2026 Aug 5;198(9):916. doi: 10.1007/s10661-026-15684-5 (PMC13442501; doi:10.1007/s10661-026-15684-5)
Supplement: Supplementary file 3 [file 10661_2026_15684_MOESM4_ESM.pdf]

Areas where soil depth profiles were obtained

Š. Tůmová[1,2], T. Matys Grygar[1]

[1] Institute of Inorganic Chemistry, Czech Academy of Sciences, 250 68 Řež, Czech Republic

[2] Faculty of Environment, J. E. Purkyně University in Ústí nad Labem, Pasteurova 3632/15, 400 96 Ústí nad Labem, Czech Republic

Supplementary Material SM4 to paper "Revisiting estimates of diffuse contamination of agricultural soils by Pb" by T. Matys Grygar et al.

Fig. S4.1 to S4.4 show sites where soil depth profiles were acquired in aim to distinguish topsoil accumulation of Pb and analyse the background Pb concentrations in varying bedrock geology. Overlap of close sites is due to preferential sampling pairs of soils under forest cover and grasslands. Fig. S4.1 and S4.2 shows areas in the northern part of the Čech Republic from the České Středohoří Mountains through Lužice and Jizera mountains to the Krkonoše Mountains, including the Pb hotspots of contamination by glassmaking discussed in Supplementary Material SM1. Třebíč Area (Fig. S4.3) and Novohradské Mountains (S4.4) are in a less Pb contaminated southern part of the Czech Republic.

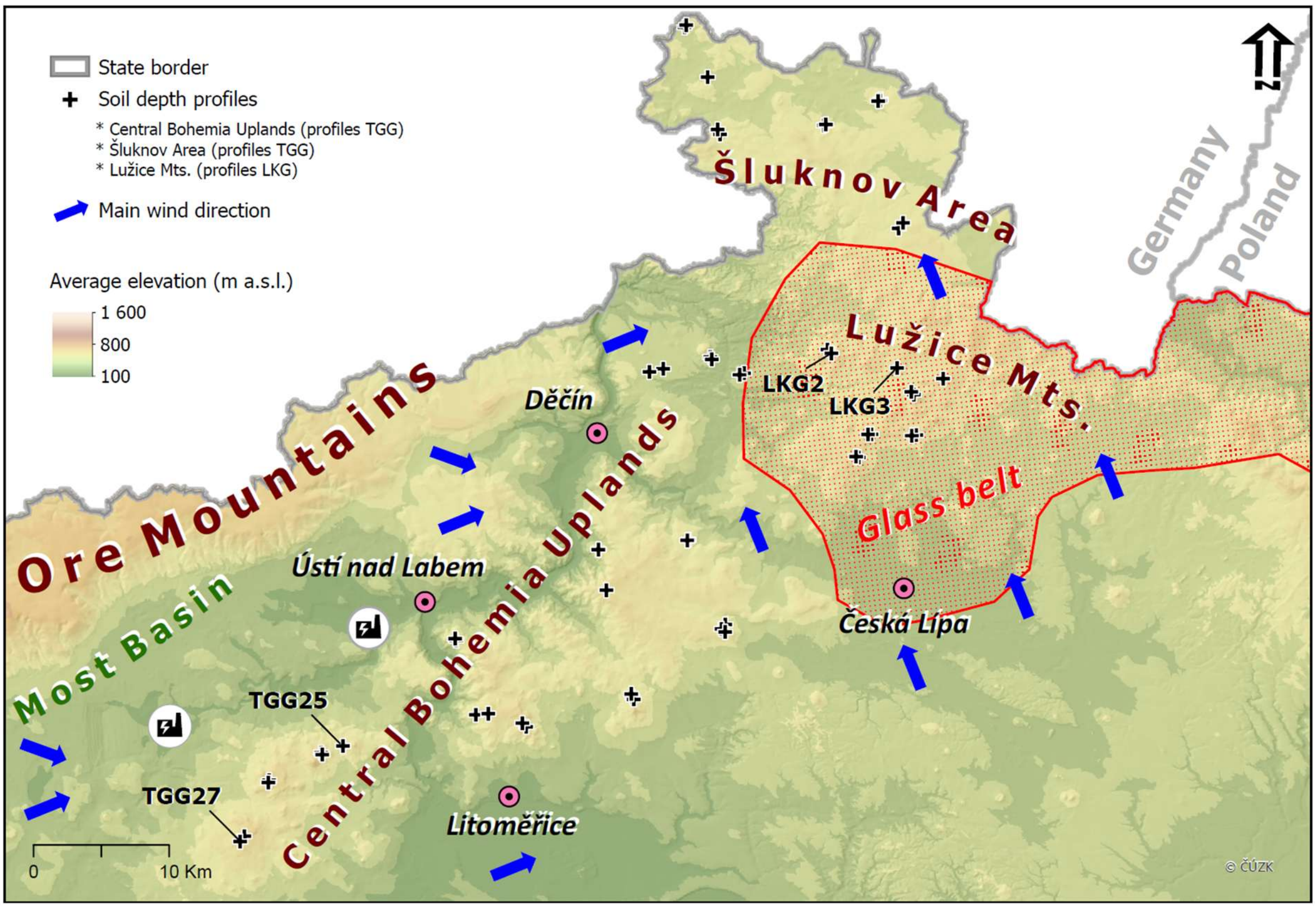

Figure S4.1. Area between coal-fuelled power plants and "glass belt".

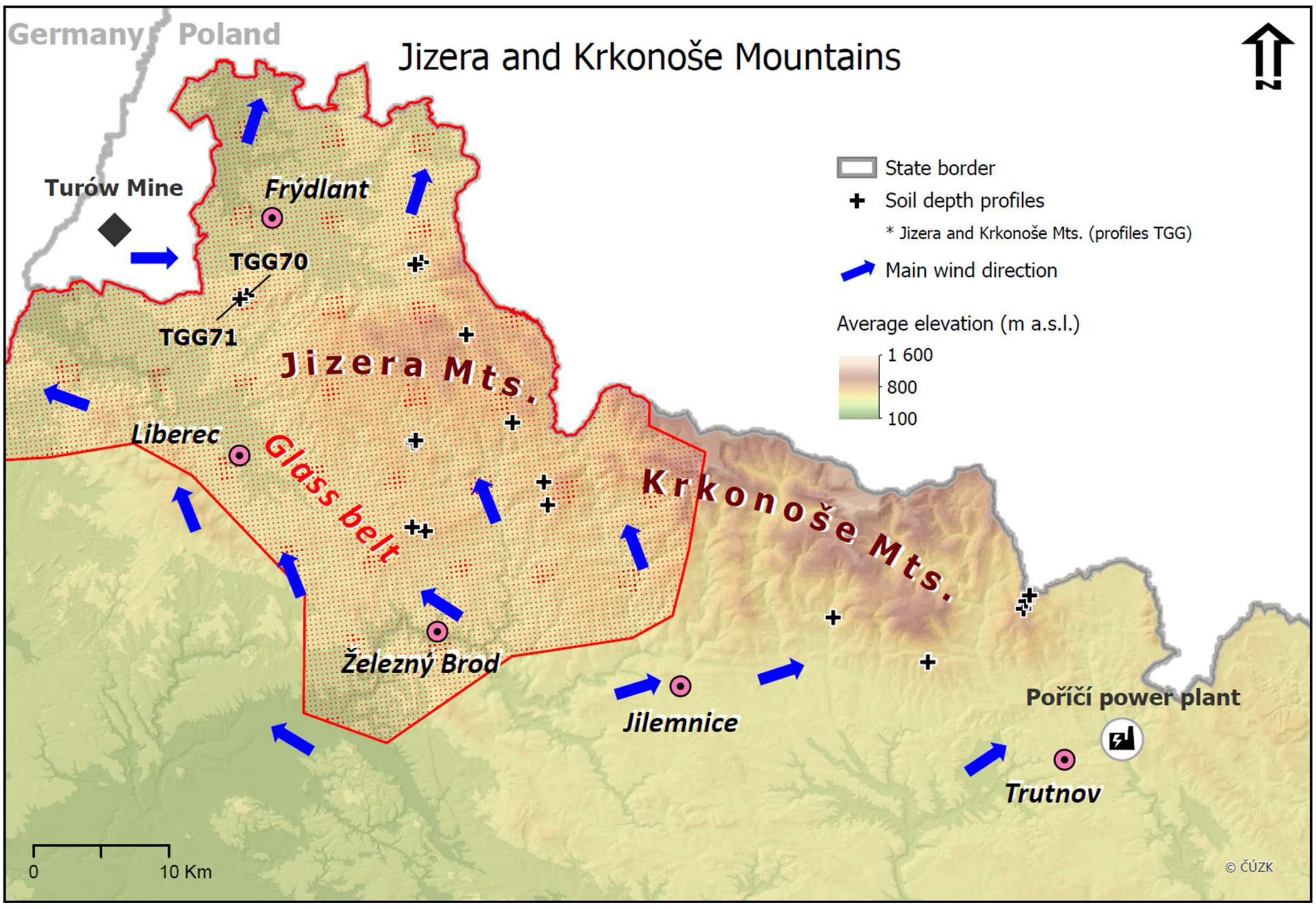

Figure S4.2. Area of the Jizera and Krkonoše mountains impacted by glassmaking and LRTC.

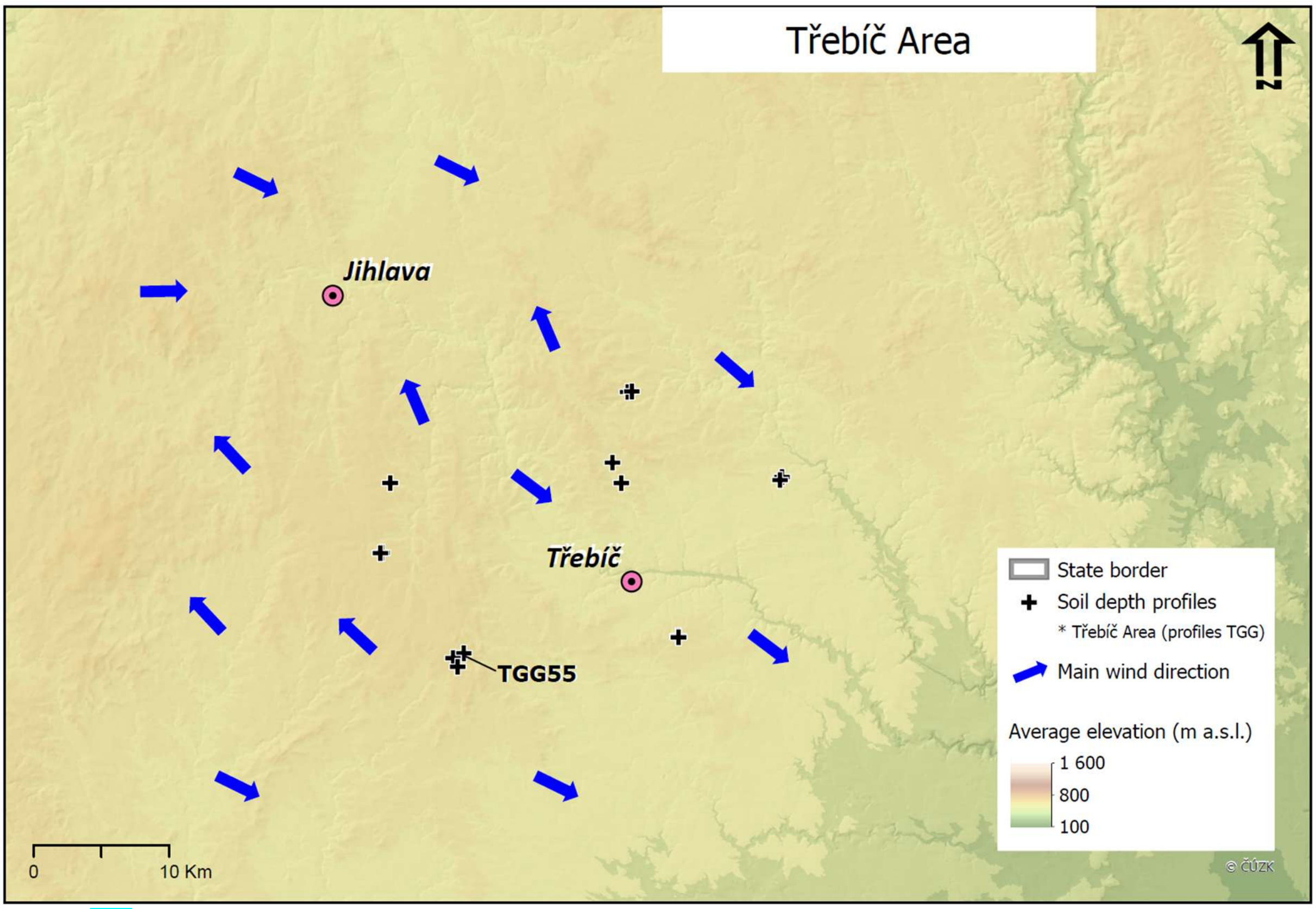

Figure S4.3. Třebíč Area – under weak influence of historical Ag smelting in Jihlava.

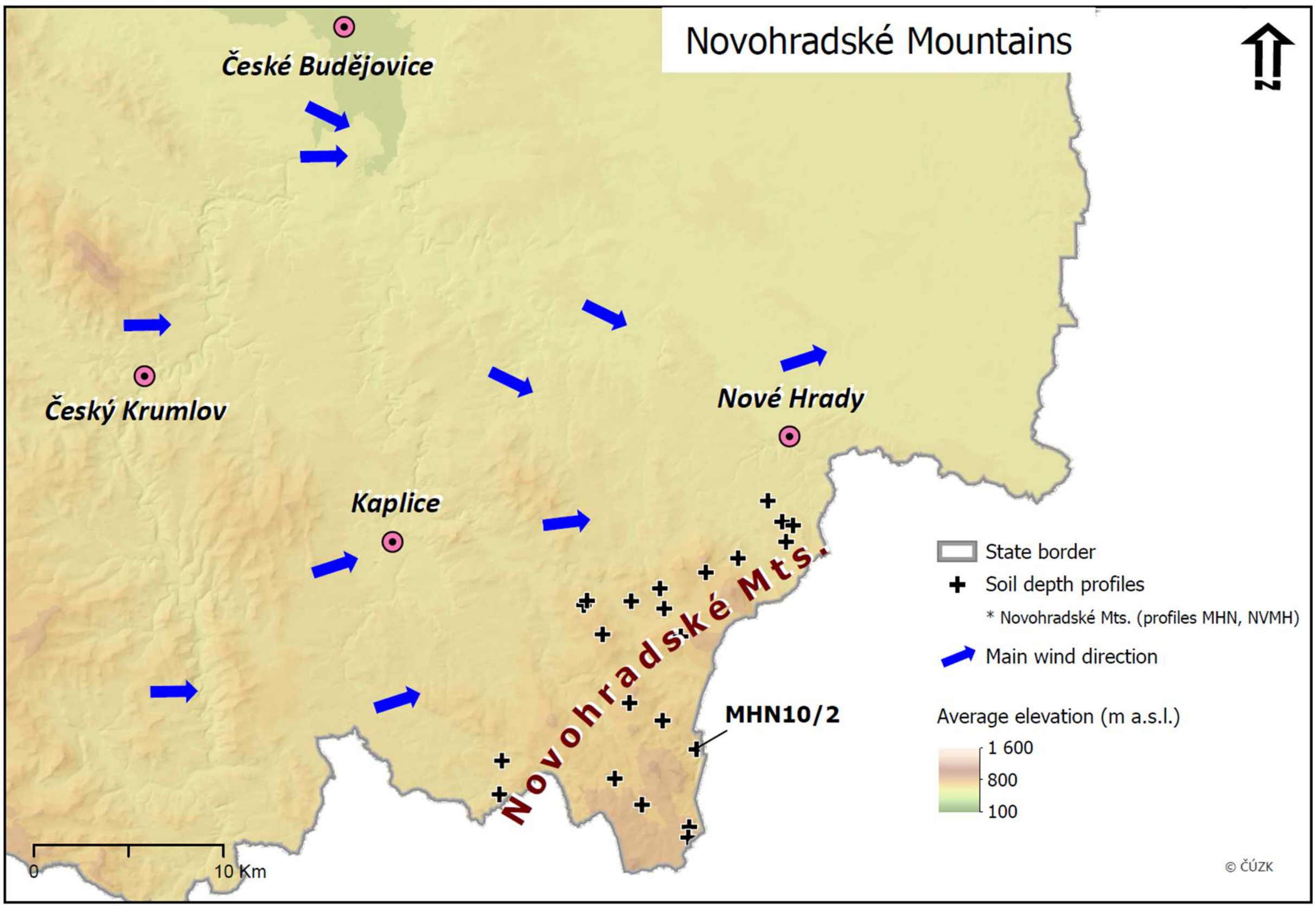

Figure S4.4. Novohradské Mountains rather remote from large historical contamination sources. Minor Ag smelting was performed in České Budějovice.
