# Supplementary material for: Revisiting estimates of diffuse contamination of agricultural soils by Pb
Source: Environ Monit Assess. 2026 Aug 5;198(9):916. doi: 10.1007/s10661-026-15684-5 (PMC13442501; doi:10.1007/s10661-026-15684-5)
Supplement: Supplementary file 4 [file 10661_2026_15684_MOESM5_ESM.pdf]

Depth profiles of total Pb in selected soil cores

M. Hošek[1,2], T. Matys Grygar[1]

[1] Institute of Inorganic Chemistry, Czech Academy of Sciences, 250 68 Řež, Czech Republic

[2] Faculty of Environment, J. E. Purkyně University in Ústí nad Labem, Pasteurova 3632/15, 400 96 Ústí nad Labem, Czech Republic

Supplementary Material SM5 to paper "Revisiting estimates of diffuse contamination of agricultural soils by Pb" by T. Matys Grygar et al.

The reliability of soil profiles was evaluated by examination of depth profiles of total Pb concentrations. From the principles of fallout impact, stratigraphically reliable soil should exhibit a simple upward increase of soil Pb concentrations as well as fallout radionuclides ^{137}Cs and $^{210}Pb_{xs}$. Depth profiles also show Al/Si ratio and Fe concentration), typically showing relative contribution of fine particles of clay and silt (higher Al/Si and Fe) and sand (usually mainly quartz, low Al/Si and Fe). The position of hereafter shown depth profiles in maps is shown in Supplementary Material SM4.

The depth profiles clearly distinguish undisturbed soil profiles from forests with quite sharp upward increase of Pb concentrations and fallout radionuclides. One such profile is shown in Fig. SM4.1 taken in TGG27 east of the Most Basin with coal-fuelled power plants. The background Pb and fallout radionuclides below determination limit were detected in the deepest sample at ca. 55 cm; all emissions are increasing upward.

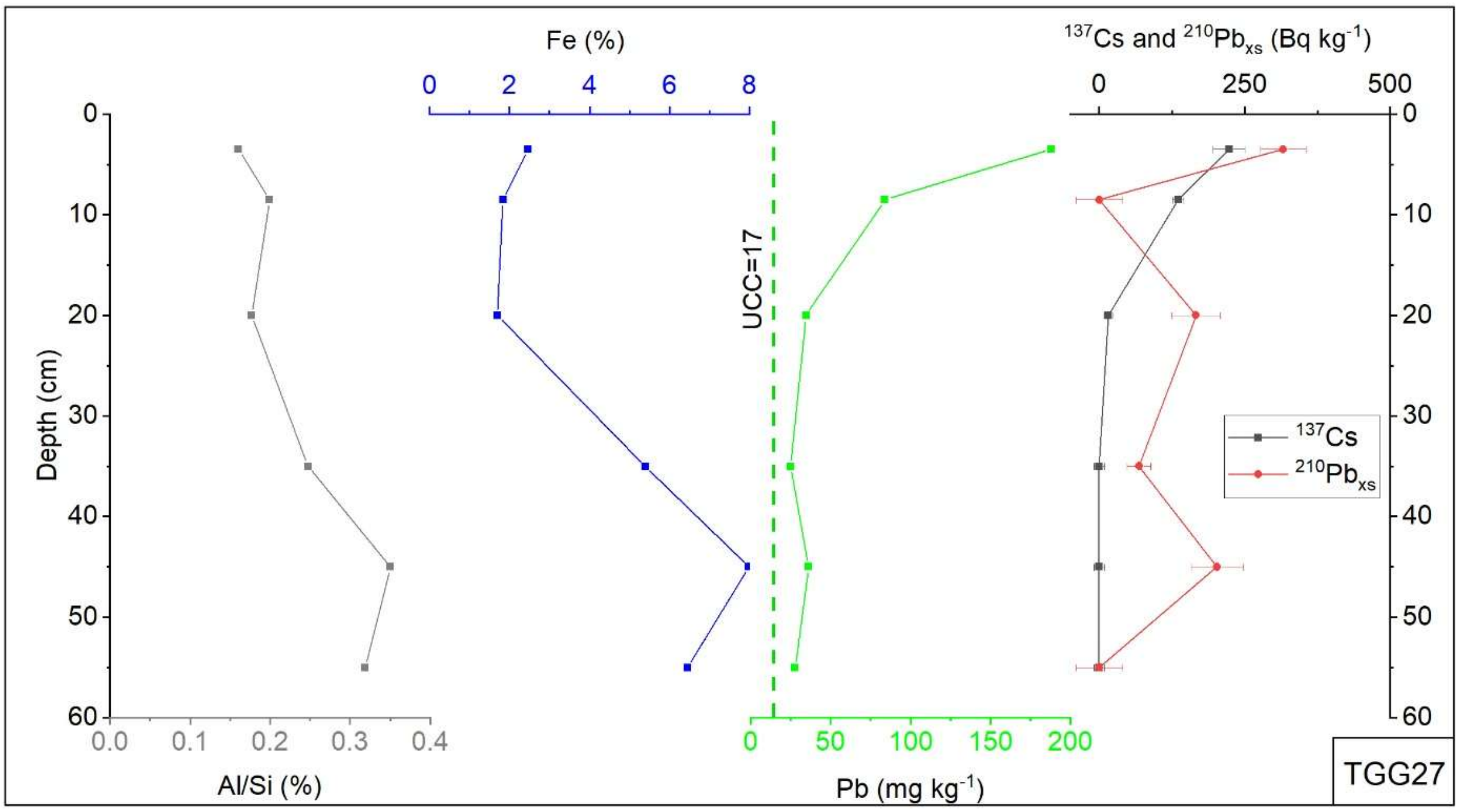

Figure S5.1. Weakly contaminated forest soil profile TGG27 from the České Středohoří Mountains.

TGG70 (Fig. S5.2) and LKG3 (Fig. S5.3) are extremely strongly contaminated forest soil profiles from the Jizera and Lužice mountains, respectively. Total Pb concentrations and both fallout radionuclides' activities increase upward to the peak in the topmost sample (TGG70) and at depth of ca. 10 cm (LKG3). The latter pattern (LKG3) is consistent with mainly historical Pb emissions and down-profile wash of ^{137}Cs known for forest topsoils mostly consisting organic matter. Noteworthy are also very high topsoil activities in TGG70 and LKG3 relative to other profiles, such as LKG2 (Fig. S5.4) close to LKG3. Some forest profiles have probably particularly effectively trapped emission loads. Generally, forest floor organic matter, originally in loose material of the O horizon, is probably relocated over the soil surface before incorporation into F+H horizons, which were sampled in this work.

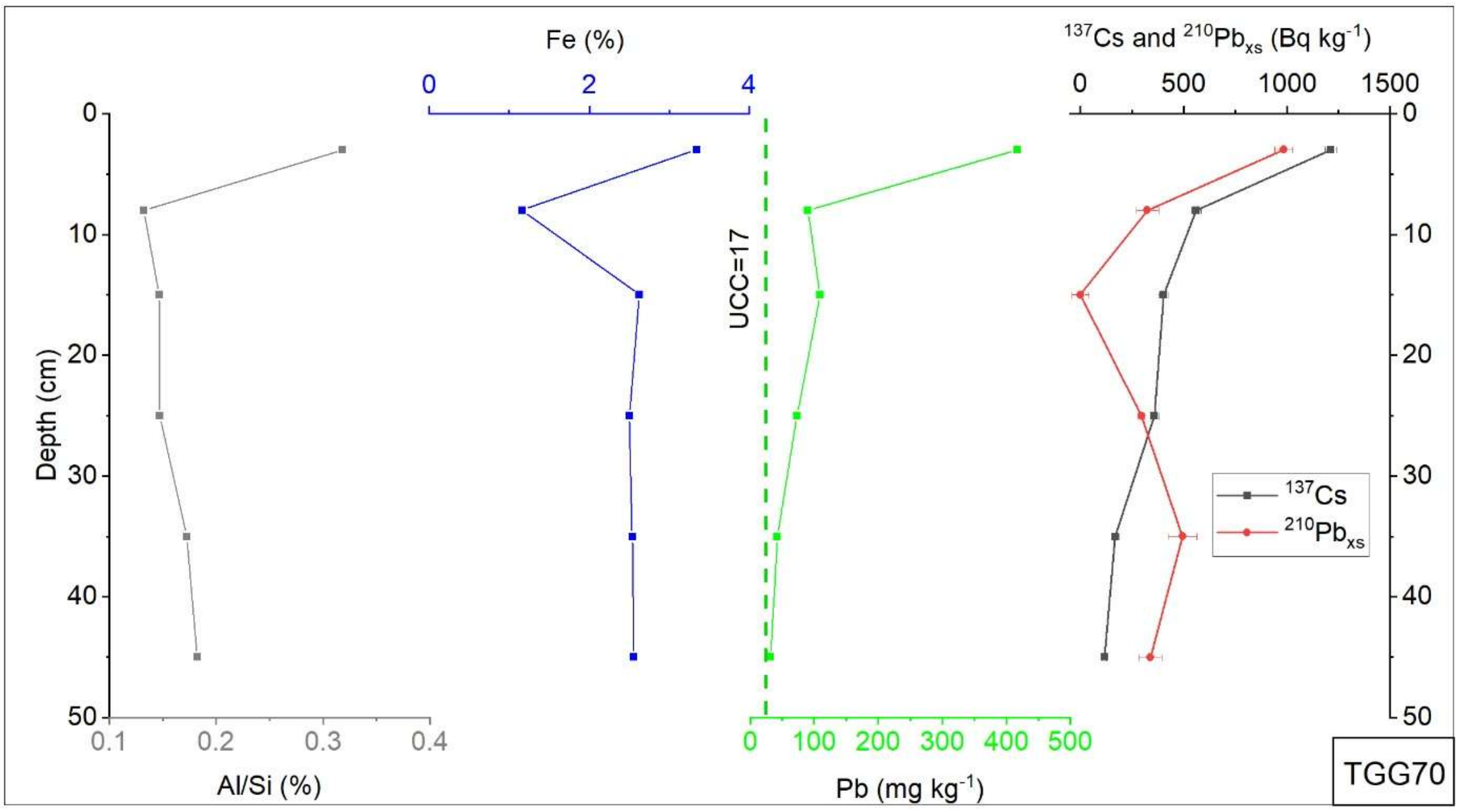

Figure S5.2. Strongly contaminated soil profile TGG70 from the Jizera Mountains.

Some soil profiles have more complex stratigraphy. In LKG2 (Fig. S5.4), the mineral subsoil (55-110 cm, uncontaminated by Pb, high Fe content) is overlain by mineral matter-poor organic topsoil (low Fe), of which the uppermost 20 cm is contaminated by Pb and fallout radionuclides. An anomalous profile on agricultural soils is shown in Fig. S5.5. The Pb maximum is not on the surface but at a depth of 10-15 cm, while fallout radionuclides are found in the top 20 cm.

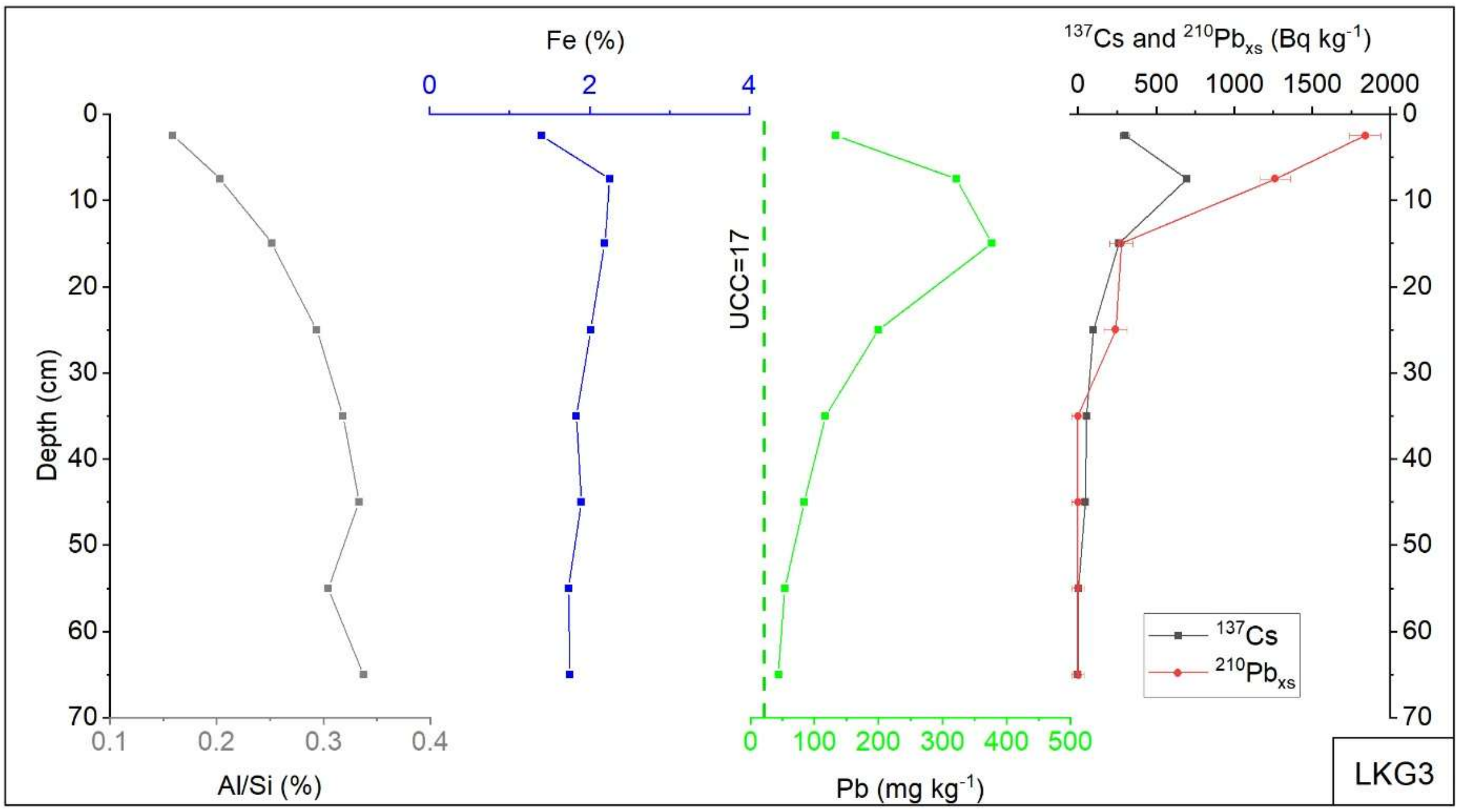

Figure S5.3. Strongly contaminated profile LKG3 from the Lužice Mountains contaminated by decorative glass making.

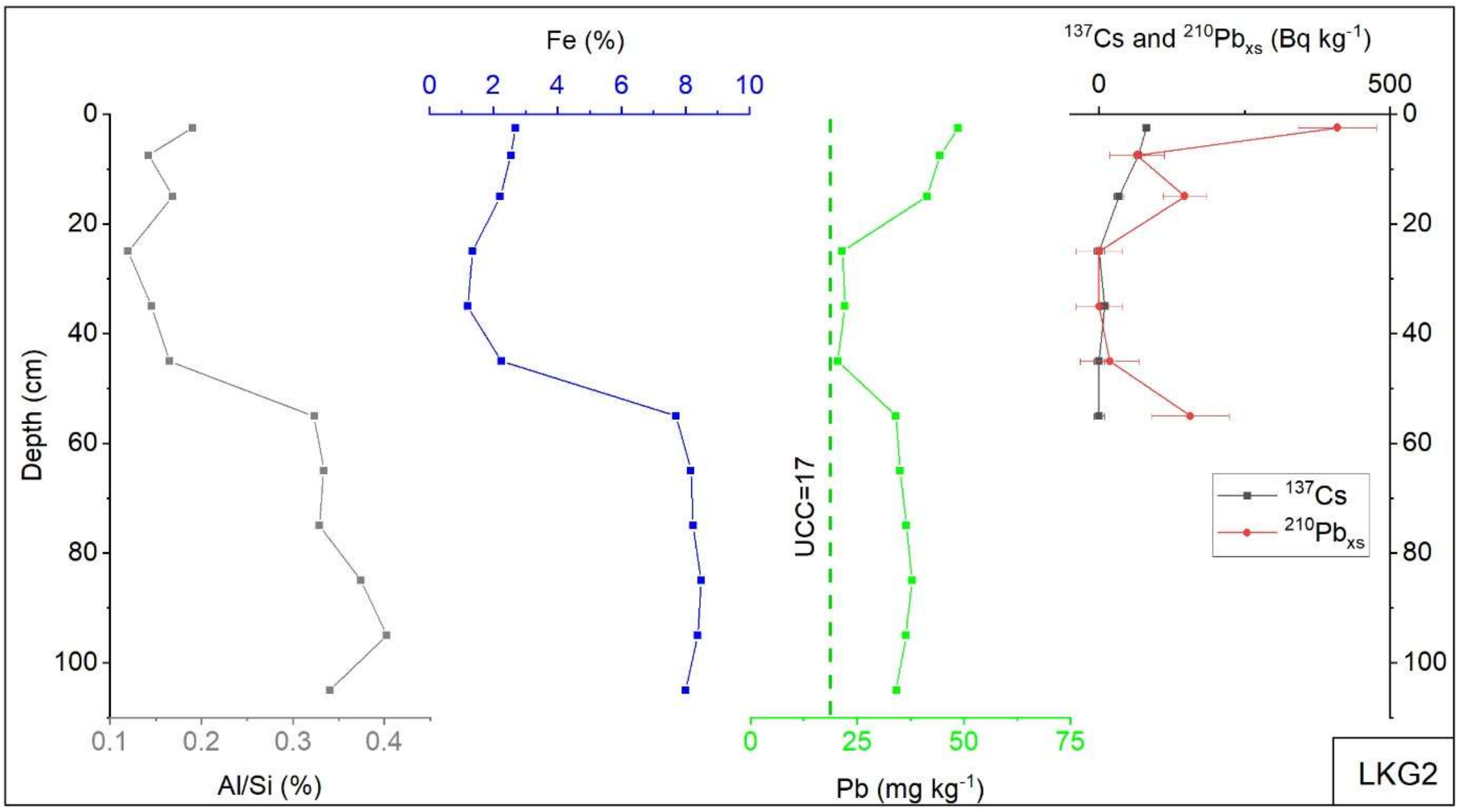

Figure S5.4. Strongly contaminated forest soil profile LKG2 with a more complex stratigraphy.

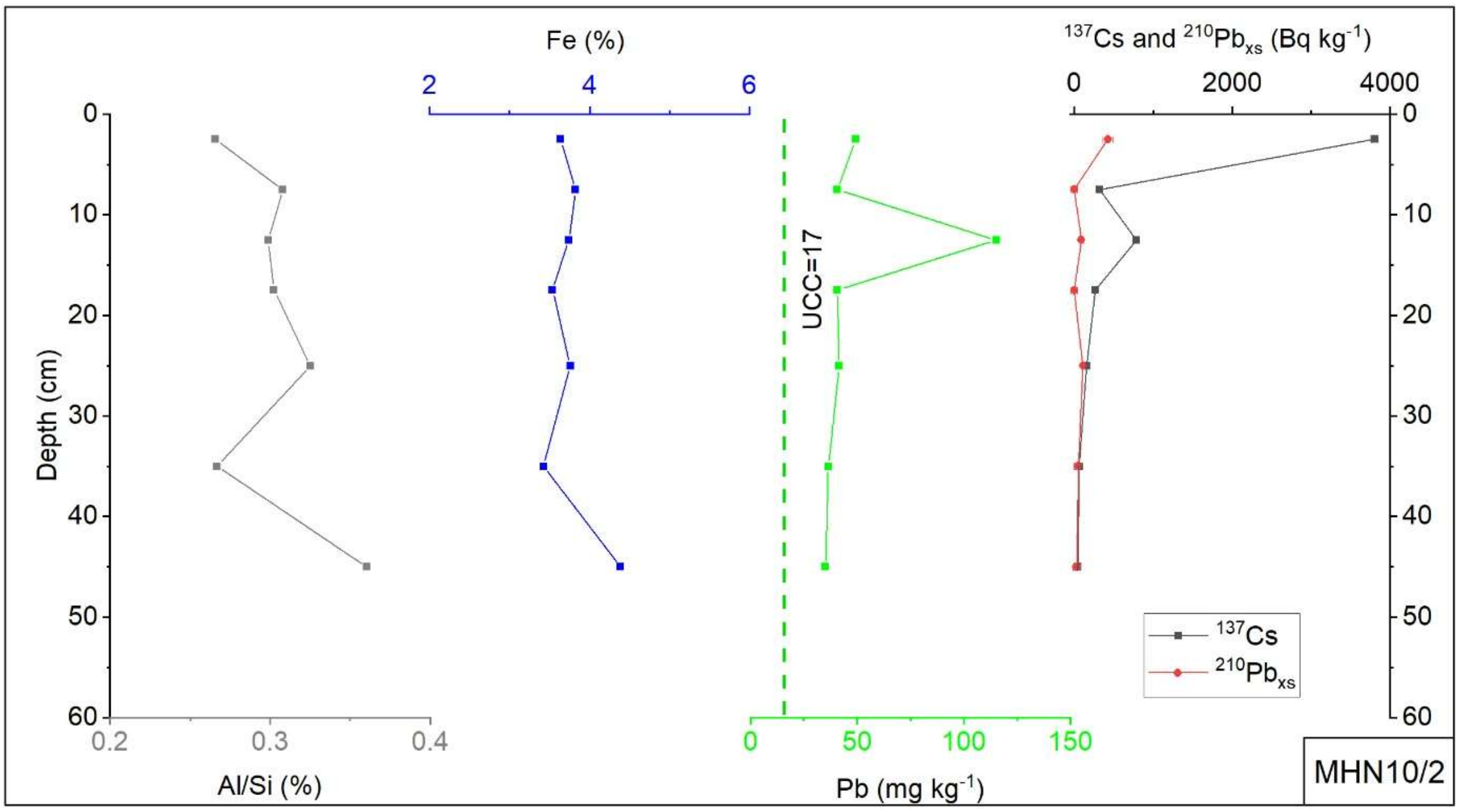

Figure S5.5. Meadow (grassland) profile MHN10/2 from the Novohradské Mountains with a more complex stratigraphy.
